# A fine kinetic balance of interactions directs transcription factor hubs to genes

**DOI:** 10.1101/2024.04.16.589811

**Published:** 2024-04-16

**Authors:** Apratim Mukherjee, Samantha Fallacaro, Puttachai Ratchasanmuang, Joseph Zinski, Alan Boka, Kareena Shankta, Mustafa Mir

**Affiliations:** 1 Department of Cell and Developmental Biology, Perelman School of Medicine, University of Pennsylvania, Philadelphia, PA 19104;; 2 Center for Computational and Genomic Medicine, Children’s Hospital of Philadelphia, Philadelphia, PA 19104;; 3 Developmental, Stem Cell, and Regenerative Biology Graduate Group, Perelman School of Medicine, University of Pennsylvania, Philadelphia, PA 19104;; 4 Howard Hughes Medical Institute, Children’s Hospital of Philadelphia, Philadelphia, PA 19104;; 5 Biochemistry and Molecular Biophysics Graduate Group, Perelman School of Medicine, University of Pennsylvania, Philadelphia, PA 19104, USA;; 6 Roy and Diana Vagelos Program in Life Sciences and Management, University of Pennsylvania, Philadelphia, PA 19104, USA;; 7. Epigenetics Institute, University of Pennsylvania Perelman School of Medicine, Philadelphia, PA 19104

## Abstract

Eukaryotic gene regulation relies on the binding of sequence-specific transcription factors (TFs). TFs bind chromatin transiently yet occupy their target sites by forming high-local concentration microenvironments (hubs and condensates) that increase the frequency of binding events. Despite their ubiquity, such microenvironments have been difficult to study in endogenous contexts due to technical limitations. Here, we overcome these limitations and investigate how hubs drive TF occupancy at their targets. Using a DNA binding perturbation to a hub-forming TF, Zelda, in *Drosophila* embryos, we find that hub properties, including the stability and frequencies of associations to targets, are key determinants of TF occupancy. Our data suggest that the targeting of these hubs is driven not just by specific DNA motif recognition, but also by a fine-tuned kinetic balance of interactions between TFs and their co-binding partners.

## INTRODUCTION

Eukaryotic gene regulation is orchestrated by sequence-specific transcription factors (TFs) that find and selectively bind DNA sequence motifs and regulate transcriptional activity in concert with a multitude of binding partners and cofactors. The ability to measure TF-chromatin interaction kinetics using single-molecule tracking within live cells and embryos has revealed that most TFs exhibit surprisingly short residence times on chromatin, on the order of just tens of seconds or less (1–4). These short residence times raised a kinetic conundrum regarding how transcription factors are able to robustly occupy their sites to drive minutes-long bursts of transcriptional activity, especially at low nuclear TF concentrations, where binding frequencies to any given site are expected to be lower than at higher nuclear concentrations. A resolution to this conundrum has come from a series of observations suggesting that instead of target occupancy being regulated by the stability of DNA binding, target occupancy may be dominated by increasing the frequency of binding events for many TFs (5–7). This increase in frequency is achieved by accumulating TFs around their targets in high local-concentration microenvironments referred to as condensates (implying liquid-like properties and phase separation based formation), or hubs, clusters, or foci when the mechanisms of formation are unknown (5, 8–14).

The formation of transcriptional hubs and condensates is often driven by selective but low-affinity interactions between intrinsically disordered regions (IDRs) of TFs, outside of their sequence-specific DNA binding domains (DBDs) (14–17). Canonically, TFs have been described as modular, with a DBD that dictates target selectivity and disordered activation or interaction domains (18–20). Yet, recent work suggests that DBDs and IDRs synergistically confer target site selection and occupation (6, 21, 22). However, the mechanisms by which the IDR-mediated localization of hubs to their target sites is conferred is unknown. Our limited understanding stems from the fact that most studies on condensate formation are performed in exogenous or overexpressed contexts, or due to technical limitations, are focused on more stable assemblies which form at specific genomic loci. Here we sought to overcome these limitations by studying hub targeting and function in an endogenous context.

We and others have previously described transient multifactor hubs formed by the pioneer transcription factor Zelda in *Drosophila* embryos (8, 23). Zelda facilitates the binding of a majority of developmental TFs to activate thousands of genes during zygotic genome activation in *Drosophila* embryos (24–26). Incorporation into Zelda hubs allows morphogenic TFs to robustly occupy target sites even at low nuclear concentrations (5, 8, 27). To investigate how Zelda hubs target specific genes, we generated embryos homozygously expressing Zelda with a mutated DBD that abolishes its recognition of its canonical DNA motifs in the embryo. We find that although this mutation diminishes the duration of individual Zelda molecules interactions with chromatin, the mutant Zelda now functionally occupies new genomic sites, driving increased accessibility and expression. Through live embryo imaging using lattice light-sheet microscopy and single-molecule tracking, we find that this differential occupation of the Zelda DBD mutant is driven by the formation of longer-lived hubs, which interact more frequently and persistently with the new target sites. We conclude that these longer-lived and more frequent hub interactions enable robust target occupancy by compensating for the reduced interaction time of individual mutant Zelda molecules with chromatin. Based on comparative analysis of the genomic localization of Zelda’s co-binding partners which themselves relocate upon Zelda deletion, we posit that Zelda hub targeting is dictated by a fine-tuned kinetic balance of interactions between site-specific binding partners. Together our results provide direct evidence for the role of hubs in regulating TF site selection, occupancy and transcriptional activity independently of a TFs canonical DNA motif recognition.

## RESULTS

### A Zelda DBD mutant functionally relocalizes to new genomic targets

**Fig. 1.**
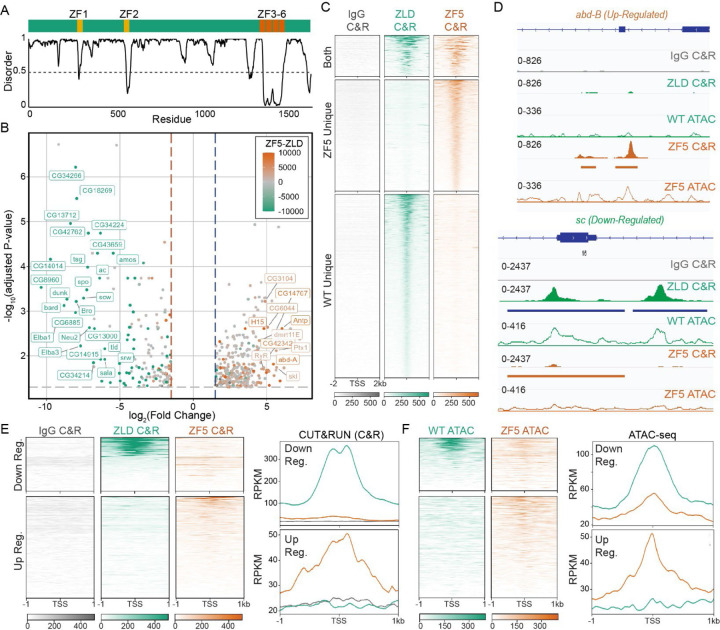
A Zelda DNA binding mutant functionally relocalizes to new genomic targets. **(A)** Domain map of Zelda indicating its DNA Binding Domain (ZF3-6). Bottom panel shows predicted disorder score (from Metapredict (28)) as a function of position. **(B)** Volcano plot of genes up- and down-regulated in the ZF5 mutant measured via RNA-seq. Points are colored by the difference in CUT&RUN (C&R) reads between ZLD and ZF5 within 2kb of the TSS. Genes with an adjusted P-value <0.01, Log_2_ Fold Change > 4, and an absolute difference in RPKM > 5000 are labeled. The gray dotted line marks adj. P-value = 0.05, blue and red dotted lines are +/−1.5 Fold Change. **(C)** Heatmap of ZLD and ZF5 CUT&RUN peaks at TSSs separated by whether they are present in WT only (2262 genes), ZF5 only (1478 genes), or both (653 genes). **(D)** Example ZLD and ZF5 CUT&RUN tracks (solid), ATAC (hollow), and called peaks (solid bars below tracks) of up- and down-regulated genes from panel B. **(E)** Heatmaps and average profiles of CUT&RUN reads around the TSSs of differentially up and down regulated genes. **(F)** Heatmaps and average profiles of ATAC-seq reads around the TSSs of differentially up- and down-regulated genes.

Zelda is maternally deposited in *Drosophila* embryos as a 1596 amino acid protein composed of a cluster of four C_2_H_2_ zinc-fingers that comprise its DNA binding domain (ZFs 3–6), and two upstream zinc-fingers (ZFs 1–2) with the remainder of it predicted to be intrinsically disordered (28) (Fig. 1A). The upstream zinc-fingers (ZFs 1–2) are not critical for Zelda’s site-specific targeting or gene activation roles in the embryo (29). Mutating the two zinc-binding cysteines in ZF5 to serines abolishes Zelda’s ability to recognize its canonical motifs in Electromobility Shift Assays (30). We generated germline clone embryos that homozygously express the ZF5 mutant Zelda and used western blots to verify that no wildtype Zelda is expressed in these embryos (Fig. S1). This line is hereafter referred to as ZF5. We generated ZF5 lines with a mNeonGreen fluorescent-protein tag (mNG-ZF5) for volumetric imaging or a mEos3.2 tag (mEos3.2-ZF5) for single-molecule tracking experiments.

To assess the functional effects of this DNA binding domain mutation we performed RNA-seq and CUT&RUN in wildtype and ZF5 embryos (Figs.1 and S2, Tables S1–S3). Consistent with Zelda’s role as an activator, we found a large number of down-regulated genes in ZF5 embryos (Figs. 1 and S3, Table S3)(26, 31). However, we were intrigued to also find a set of significantly up-regulated genes in ZF5 embryos (Fig. 1B) of which only 2.4% are also upregulated in Zelda null embryos (26) (Fig. S3). This differential activity suggested that the genes up-regulated in ZF5 embryos might be due to a relocalization of ZF5 to these genes. Consistent with this hypothesis, CUT&RUN in WT and ZF5 embryos showed that ZF5 localizes to the Transcription Start Sites (TSSs) and gene bodies of up-regulated genes (Fig. 1D–E). Conversely, ZF5 is no longer present at the TSSs and gene bodies of genes that are down-regulated in ZF5 embryos. We found a 2.1-fold increase in binding at the TSSs (over a 80bp window) of up-regulated genes and a 9.2-fold decrease in Zelda binding at down-regulated genes (Fig. 1E). To test if ZF5s relocalization could also drive increased accessibility, we performed ATAC-seq on WT and ZF5 embryos and found a 2-fold increase in accessibility at up-regulated genes and a 2-fold decrease at down-regulated genes (Fig. 1D,F, Fig. S4 and Table S3). Contrary to recent observations on regions outside of a TFs DBD driving site-specific occupation at canonical target sites (21, 32), we found that the ZF5 mutation causes Zelda to broadly relocalize to new target sites, which we hypothesize is through a combination of protein-protein and protein-chromatin interactions.

**Fig. 2.**
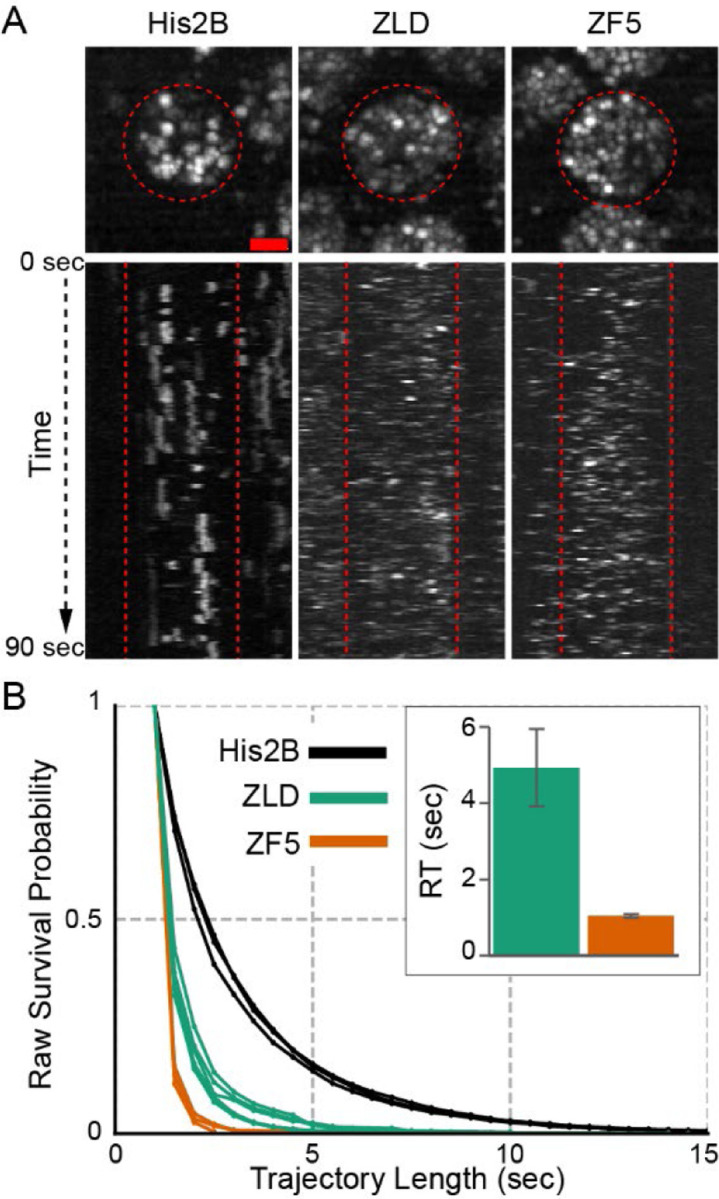
Mutation to Zelda’s DNA Binding Domain destabilizes its interaction with chromatin. **(A)** Maximum intensity projections over 90 seconds of raw tracking data. Red circles indicate nuclear boundaries. Kymographs (x-t maximum projections) of single molecule detections of mEos3.2-ZLD, mEos3.2-ZF5, and H2B-mEos3.2. Acquired with an exposure time of 500 msec, scale bar is 2 μm. **(B)** Raw survival probabilities of single molecule trajectories from at least 3 individual embryos for mEos3.2-ZLD, mEos3.2-ZF5, and H2B-mEos3.2. Inset shows residence times, after correction for photobleaching, for mEos3.2-ZLD (4.93±1.01 sec) and mEos3.2-ZF5 (1.04±0.05 sec).

To understand how the perturbation to Zelda’s DBD alters its interaction kinetics with chromatin, we used single-molecule tracking to measure its residence time on chromatin. As previously described, we used long exposure times of 500 msec to blur fast-diffusing molecules into the background and minimize photobleaching (5, 8, 33–35). We collected single-molecule data in live embryos expressing either mEos3.2-ZLD, mEos3.2-ZF5, or H2B-mEos3.2 (Movie 1). A loss of stably associated trajectories is apparent in mEos3.2-ZF5 compared to mEos3.2-ZLD both visually (in kymographs, Fig. 2A) and in the quantification of survival probabilities (Fig. 2B). By fitting the H2B-mEos3.2 data to estimate the rate of photo-bleaching and de-focalization, we determined the apparent residence time of ZLD and ZF5 on chromatin and found that it is significantly reduced upon the DBD mutation from ~5 seconds to just ~1 second (Fig. 2B), confirming that ZF5 is significantly impaired in its ability to bind chromatin. Together, the genomics and residence time data suggest that despite its impaired ability to stably bind DNA, ZF5 can still occupy genes and drive expression. We reasoned that the altered targeting of ZF5 could provide insights into general mechanisms of TF target specificity and decided to explore this idea through a combination of live imaging and further genomics analyses.

### Perturbation to Zelda’s DBD alters its target search strategy

**Fig. 3.**
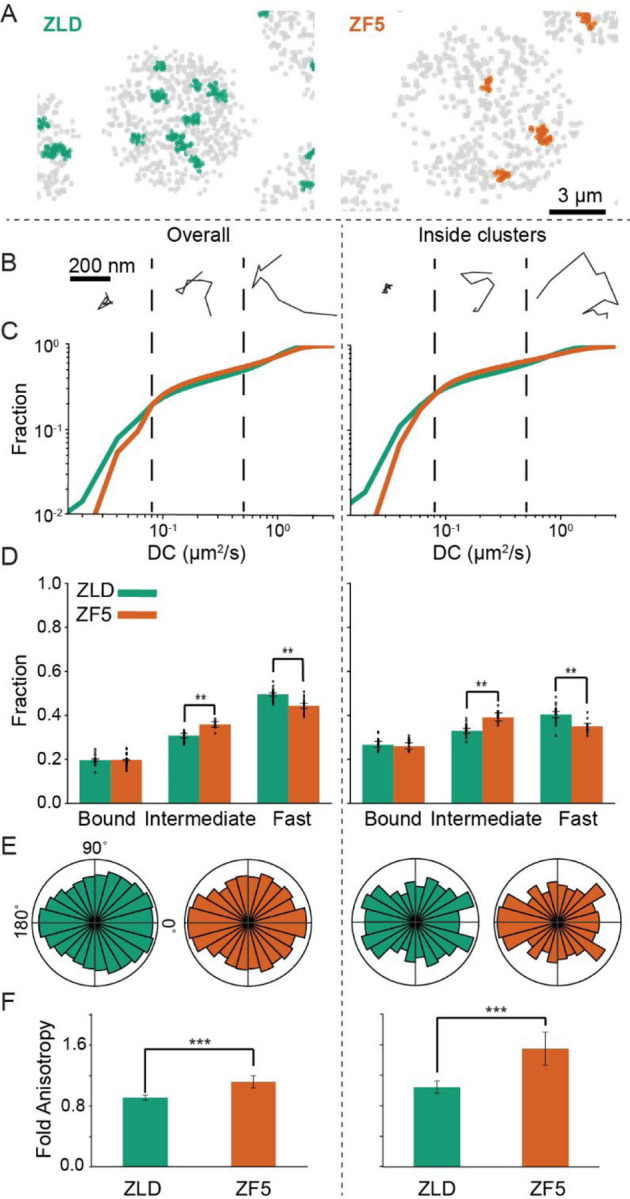
Perturbation to DNA binding domain alters Zelda’s nuclear exploration kinetics. (A) Representative maps of average trajectory positions (in gray) in nuclear cycle 14 from tracks accumulated over 80 sec at 10 msec/frame. Clusters are marked in green for ZLD and orange for ZF5. (B) Example trajectories from each kinetic category. (C) Cumulative distribution of diffusion coefficients (DC) for ZLD and ZF5 overall (left) and inside clusters (right) (n= 70,827 tracks overall and 14,156 in clusters for ZLD and n=40,307 overall and 14,516 in clusters for ZF5). Black dashed lines are cutoffs for bound, intermediate, and fast trajectories. (D) Bound, intermediate, and free fractions overall (left) and inside clusters (right). Error bars represent standard deviations from bootstrapping analysis. Points show the fractions from 23 and 14 individual ZLD and ZF5 movies respectively, from at least 3 embryos each. (E) Distribution of angles between two track segments for non-bound trajectories overall (left) and in clusters (right). (F) Fold anisotropy overall (left) and in clusters (right). n=9,806 overall and 1,673 in clusters for ZLD and 4,609 overall and 995 in clusters for ZF5. Error bars represent standard deviations from bootstrapping analysis.

To quantify how reduced chromatin binding times alter Zelda’s diffusion kinetics, we performed fast single-molecule tracking (10 msec/frame) on mEos3.2-ZLD and mEos3.2-ZF5 (Movies 2 and 3) in homozygously expressing embryos. Visual examination of single-molecule tracks shows that both ZLD and ZF5 exhibit a range of kinetic behaviors (Fig. S5A) consistent with previous observations on ZLD (8). Both ZLD and ZF5 tracks are spatially clustered, though the number of detected clusters is reduced in ZF5 embryos (Fig. 3A and Fig. S5B). To compare diffusion kinetics inside and outside clusters, we used a Baysian analysis method (SASPT, (36)) to generate a probability density over a range of diffusion coefficients (DCs) for each trajectory (Fig S5C). Based on inflection points in the calculated DC spectrum, we defined three kinetic bins of slow (DC ≤ 0.08 um^2^s^−1^), intermediate (0.08 um^2^s^−1^ <DC< 0.5 um^2^s^−1^), and fast (DC≥ 0.5 um^2^s^−1^) moving molecules (Figs. 3B–D and S5D). The slow population was inferred to be chromatin-bound based on the diffusion spectrum of His2B data (Fig. S5D). Perturbing the DNA binding domain does not alter the fraction of Zelda trajectories in the bound state, either overall (~20% for both ZLD and ZF5), or inside clusters (~25% for both ZLD and ZF5). Thus surprisingly, despite the loss in residence time, the fraction of molecules that exhibit chromatin bound-like mobility does not change in ZF5 when compared to ZLD. In contrast, the intermediate fraction is significantly higher in ZF5 than ZLD overall (36±1.2% for ZF5 vs 31±1.1% for ZLD) and inside clusters (39±0.7%, ZF5 vs 33±0.4%, ZLD). This increase in the intermediate fraction comes from a proportional loss in the fast fraction. We note that in previous analysis (8) a simpler two-state model was used to estimate a bound fraction of ~50% for ZLD which represents the sum of the bound and intermediate states reported here. The average diffusion coefficient of molecules within each bin (Fig. S5E) is not significantly different between ZLD and ZF5 allowing us to conclude that while there is a higher proportion of ZF5 molecules in an intermediate mobility state, the overall kinetics of how Zelda moves through the nucleus is not dominated by its canonical DNA binding interactions.

The increase in the intermediate kinetic population of ZF5 suggests that it explores the nucleus using a more compact search strategy (37) in which molecules spend more time constrained, or trapped, in nuclear sub-regions, often retracing their steps. To explore the differences in the search strategy between ZF5 and ZLD, we calculated the angles between three consecutive displacements in single-molecule trajectories. To avoid errors in our angle calculation and to exclude motion anisotropy resulting from chromatin interactions, we only considered molecules in the intermediate and free populations and with displacements larger than 200 nm based on the displacement distribution of His2B (Fig. S5F)(7, 37–39). The distribution of angles shows an increased diffusional anisotropy for ZF5 overall and inside clusters (Fig. 3E). We further quantified these changes in terms of fold-anisotropy, defined as the ratio of the fraction of angles falling in the 180±30° range to the 0±30° range and found a 1.2- and 1.5-fold increase in ZF5 anisotropy over ZLD overall and inside the clusters respectively (Fig. 3F). Analyzing changes in fold-anisotropy as a function of displacement length shows that although the fold-anisotropy decreases with distance for both proteins, ZF5 exhibits anisotropic behavior over longer distances than ZLD (Fig. S5G). These changes in anisotropy are consistent with Zelda exploring the nucleus in a more compact manner, meaning molecules more frequently revisit regions, after its DNA binding activity is perturbed. We hypothesized that this behavior is driven by ZF5 being more frequently trapped in hubs to facilitate its target search (7), potentially explaining its ability to occupy its new target sites despite a reduced residence time.

### DNA binding deficient Zelda forms fewer but longer-lived hubs

**Fig. 4:**
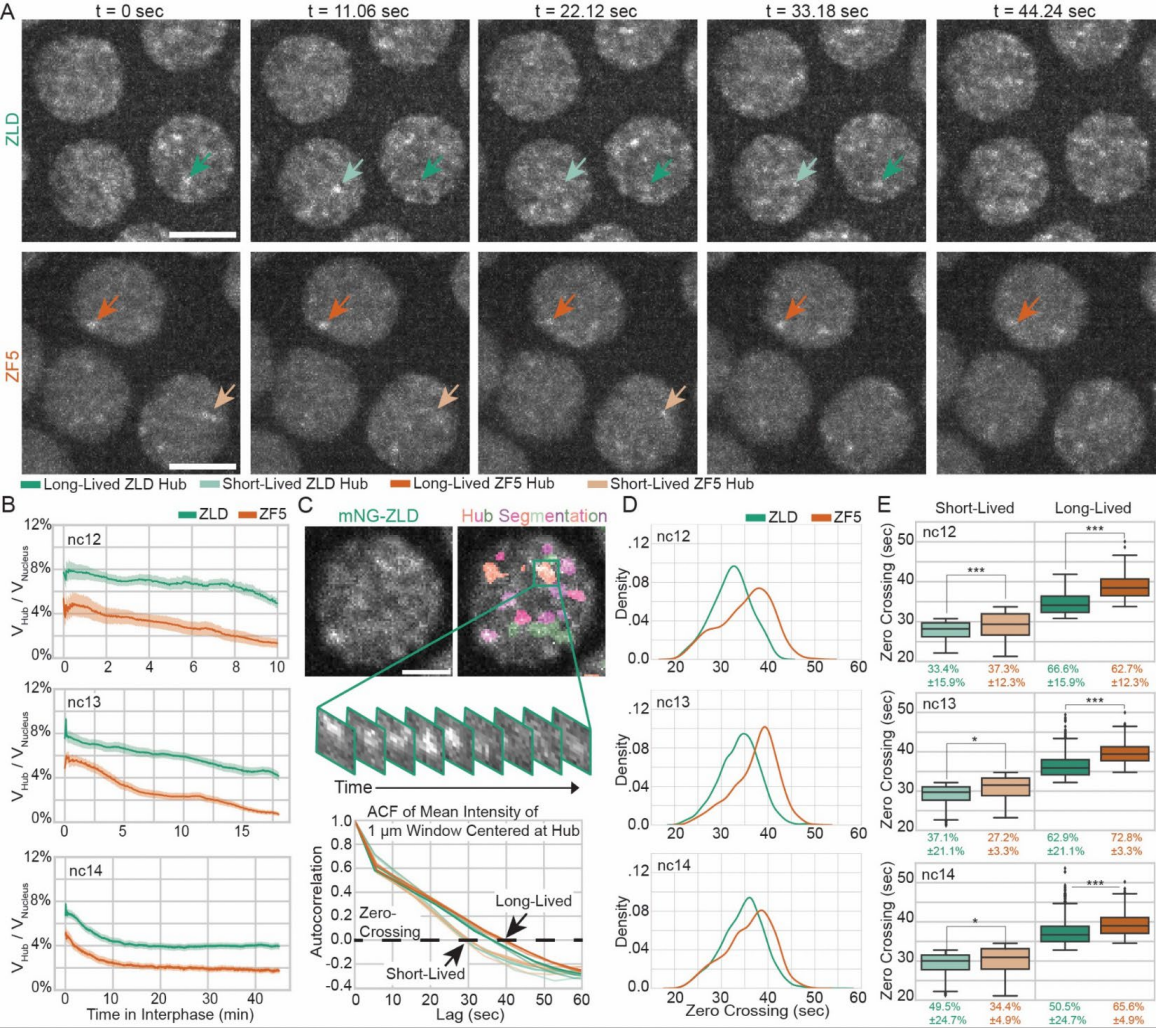
Perturbation of Zelda DBD leads to fewer but longer-lived hubs. **(A)** Single z-slices, from volumetric data, of mNeonGreen-ZLD (top) and mNeonGreen-ZF5 (bottom) over 45 seconds during nuclear cycle (nc) 13. Max projection images are shown at 11.06 second intervals from volumes acquired every 5.53 seconds. Dark green/orange and light green/orange arrows show representative long-lived and short-lived hubs respectively. Scale bars are 5 microns. **(B)** Fraction of the nuclear volume occupied by hubs in interphases of nc12, 13, and 14. For ZLD, 73 nuclei from 4 embryos in nc12, 312 nuclei from 9 embryos in nc13, and 895 nuclei in 13 embryos in nc14, and for ZF5, 341 nuclei from 5 embryos in nc12, 127 nuclei from 4 embryos in nc13, and 445 nuclei from 5 embryos in nc14 were analyzed. **(C)** Hubs were segmented and intensity autocorrelations were calculated in 1 µm^3^ boxes centered on hubs. Hubs were separated into short-lived and long-lived populations based on a two-component gaussian mixture model. Average autocorrelations for the short-lived and long-lived populations in each nc for ZLD (green) and ZF5 (orange) are shown. For lifetime analysis 346 hubs in nc12; 2,315 hubs in nc13; 5,758 hubs in nc14 for ZLD, and 598 hubs in nc12, 810 hubs in nc13, 1,770 hubs for ZF5 in nc14, were analyzed from the nuclei and embryos in B. Scale bar is 2 µm. **(D)** Probability density plots show the distribution of zero-crossings from the autocorrelations for ZLD and ZF5. **(E)** Quantification of zero-crossings and proportions of hubs in short- and long-lived states. Line inside each box represents the median value. Each condition was randomly subsampled to 100 hubs per condition for statistical testing. Significance was measured using Kruskal-Wallis test followed by Mann-Whitney U-Test for pairwise comparisons. The proportion of all hubs within short- or long-lived groupings are reported below the box-plot along with the standard deviation between embryos replicates. For ZLD, 4 embryos in nc12, 9 embryos in nc13, and 13 embryos in nc14, and for ZF5, 5 embryos in nc12, 4 embryos in nc13, and 5 embryos in nc14 were analyzed.

The increase in trapping of ZF5 molecules compared to ZLD could result from a change in the number or temporal stability of hubs. To test this idea, we acquired lattice light-sheet data over a ~20 µm thick volume of nuclei along the surface of the Drosophila embryo every 4–6 seconds throughout nuclear cycles (ncs) 12–14 in embryos homozygously expressing mNeonGreen-ZLD and mNeonGreen-ZF5 (Fig. 4A, Movies 4, 5, 6). Through visual examination of these movies, we observe that ZLD forms transient hubs that appear discrete in ncs 12 and 13, and take on a more connected appearance in nc 14. In contrast, ZF5 forms hubs that appear longer-lasting than those formed by ZLD but are fewer per nucleus. Additionally, we observe that ZF5 hubs remain discrete in nature through ncs 12–14 (Movies 4–7).

We developed a custom analysis pipeline to segment hubs (Fig. S6, Movie 10) and quantify their properties including volume fraction, enrichment, and lifetimes (Figs. 4B–E, S6–S8). We found that ZF5 hubs occupy a smaller fraction of the nuclear volume, and have lower fold-enrichment over the nuclear mean intensity due to a smaller fraction of ZF5 protein being incorporated into them (Figs. 4B and S6 B,C). To assess if wildtype ZLD interacts with ZF5 and rescues hub phenotypes, we imaged ZF5 in heterozygous embryos (one copy of mNG-ZF5 and one copy of unlabeled ZLD) (Movie 8). In heterozygous embryos, ZF5 hubs are more enriched and occupy a larger fraction of the nucleus than in homozygous ZF5 embryos, but these properties are still decreased compared to wildtype ZLD hubs (Fig. S7). These data suggest that the presence of wildtype ZLD does partially rescue ZF5 hub properties. To assess if this interaction between wildtype ZLD and the ZF5 mutant is mediated through their IDRs. We imaged both heterozygous and homozygous mNG-ΔIDR embryos. We note that unlike ZLD and ZF5 which have hubs present throughout nc12-14, the ΔIDR mutant has no visible hubs present until the end of nc13, when nuclei enter prophase, and about 15 minutes into nc14 where about 2–3 highly stable aggregate-like bodies form (Fig. S7, Movie 9). While ΔIDR embryos do not exhibit any significant hub formation through most of interphase, a greater fraction of the nuclear volume in heterozygous ZF5 embryos is occupied by hubs compared to homozygous ZF5, implicating both homo- and heterotypic IDR interactions in driving ZLD hub formation and the DBD in regulating where they form (Figs. 4B and S7).

Although the number of hubs is reduced in ZF5 embryos, we noticed that the ones that remain appear to be more stable in time (Fig. 4A and Movies 4–6). To quantify the lifetime of hubs, we performed autocorrelation analysis in a 1 μm^3^ box centered at hubs in the middle of the interphase of each nuclear cycle (Fig. 4C). This analysis revealed that both ZLD and ZF5 form two distinct populations of shorter- and longer-lived hubs with similar mean lifetimes, on the order of 30 and 40 seconds respectively(Fig. 4D). However, a greater proportion of ZF5 hubs are longer lived (50.5±24.7% for ZLD vs 65.6±4.9% for ZF5 in nc14, Figs. 4E and S8). As hubs are thought to buffer the short residence time of proteins on chromatin by facilitating an increase in binding frequency, we hypothesized that these more stable hubs drive the functional occupation of ZF5 at its new targets by more frequently localizing with them.

### DBD mutant Zelda hubs are relocalized to a differentially upregulated gene

**Fig. 5:**
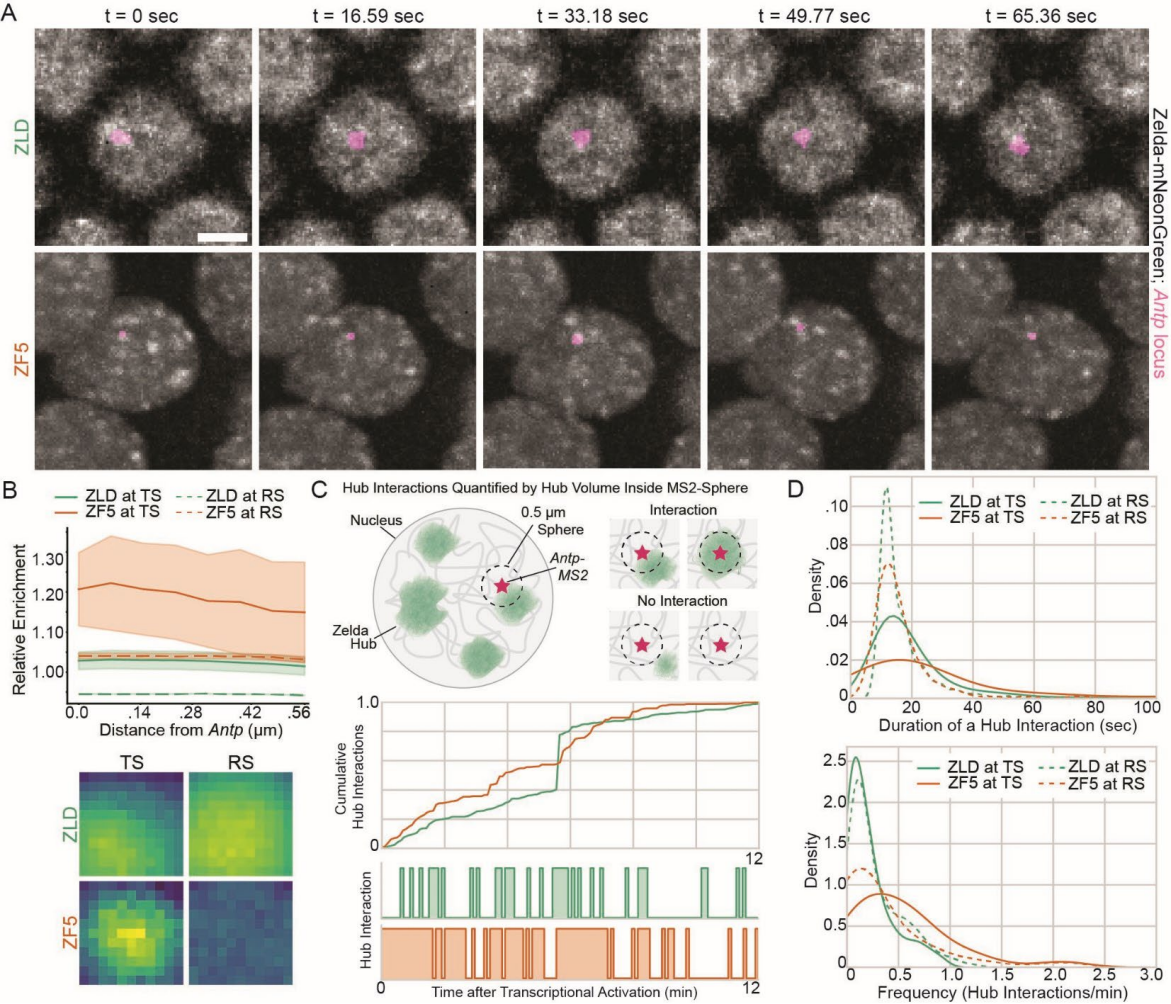
Zelda DBD mutant upregulates new targets through hub relocalization. **(A)** Images show max projections of Zelda nuclear distribution (gray) of ZLD (top) and ZF5 (bottom) in nc14 where actively transcribing *Antp* locus (magenta segmented MS2 spot) is located. Scale bar = 5 µm. Volumes were acquired every 5.53 seconds while chosen images show 16.59 second intervals. **(B)** Cropped images show average intensity centered either at the transcription site (TS) or random control site (RS) in the nucleus. Image size is 1.1 µm x 1.1 µm. All cropped images have the same contrast adjustment. Average radial profile at the *Antp* shows Zelda (ZLD or ZF5) enrichment as a function of distance from MS2 spot center. **(C)** Hub interactions are quantified using overlapping hub pixels within a 0.5 µm sphere centered at the *Antp*-MS2 spot (TS) or at randomly selected control sites (RS). Cumulative hub volumes in this sphere are quantified over time as a proxy for hub interactions where two consecutive frames with a hub volume within the MS2 sphere indicate an interaction. Representative cumulative (top) and individual (bottom) hub interaction traces from one nucleus of each condition are plotted. **(D)** Quantification of the duration in seconds of individual hub interactions and the frequency of these interactions per minute with the *Antp* locus. A Mann-Whitney U Test was performed on all pairwise relationships in the duration and frequency plots. For the duration, no significant difference was calculated between the TS for ZLD and ZF5. However, p < 0.001 between the RS for ZLD and ZF5 and between TS and RS for each of the conditions. For frequency of hub interactions, p < 0.05 for all pairwise relationships besides between RS for both conditions have no significant difference. For all panels, we analyzed 3 ZLD embryos with a total of 197 analyzed nuclei and 2 ZF5 embryos with a total of 56 analyzed nuclei in nc14.

We had previously observed that the occupancy of transcription factors at their sites is driven by transient but preferential and frequent hub interactions (8). We reasoned that ZF5’s functional relocalization to new target sites, as determined by our genomics experiments, is driven by a relocalization of hub interactions to this new site. To test this idea we performed simultaneous imaging of ZLD or ZF5 hubs and transcriptional activity using the MS2-MCP system. We selected *Antp* as the target gene of interest as it is significantly upregulated and shows increased accessibility and occupation in ZF5 embryos and not in Zelda null embryos (Figs. 1, S9, and Table S1). We observed the earliest activation of *Antp* transcription during cellularization in nc14 in wildtype embryos (Movie 11). However, in ZF5 embryos, we found that the *Antp* gene is active as early as nc12 and continues into late nc14 (Fig. S5, Movies 12–14). Visually, we did not observe any significant associations between wildtype ZLD hubs and the active *Antp* locus (Fig. 5A, Movie 11) but saw a striking apparent increase in the frequency of hub association to the transcribing *Antp* locus in ZF5 embryos (Fig. 5A, Movie 14). We quantified the differences in TF enrichment driven by hub interactions at these sites as described previously (8) and found that there is significant enrichment of ZF5 at the transcription sites compared to random sites in the nucleus which is not present in ZLD embryos (Figs. 5B, Fig. S10 and Movies 12, 13).

To further assess the kinetics of how hubs interact with the *Antp* locus, we localized and tracked MS2-MCP spots and quantified all hub interactions with the locus. As noted previously, during nc14, ZLD shows more network-like and less discrete hubs than ZF5 (Fig 4A). Due to the network-like structure, quantifying center-to-center distances of non-discrete entities proved futile. Instead of center-to-center distances, we quantified the volume of a 0.5 µm sphere centered at *Antp* taken up by hubs (Fig. 5C). To account for random hub interactions, we compared the volume fraction of hubs near *Antp* to randomly selected control spots at least 1.5 µm away from the transcription site. We measured the number of consecutive frames where hubs were proximal to the transcription site, with at least two consecutive frames (~11 sec) required to be labeled as an interaction. Using this approach, we quantified both the duration and frequency of hub interactions (Fig. 5C). We found that both the duration of individual hub interactions is increased in ZF5 (30.27±37.40 sec) compared to ZLD (20.77±21.89 sec) (Fig. 5D) and the frequency of hub interactions is increased in ZF5 embryos (0.53±0.48 interactions/min) compared to ZLD embryos (0.22±0.22 interactions/min) (Fig. 5D). In addition to the increase in mean interaction frequency values, we observe a larger spread in the distribution of the frequency of hub interactions across nuclei (variance increases from 0.05 in ZLD to 0.23 in ZF5 and skewness increases from 1.50 in ZLD to 1.71 in ZF5). For instance, over 13% of ZF5 nuclei have a frequency of at least one interaction per minute while there are no nuclei in ZLD embryos with this interaction frequency. This difference in hub interactions is stark considering that ZF5 has a significantly lower fraction of the nucleus occupied by hubs in each nucleus than ZLD (Fig. 4B), and yet *Antp* experiences more hub interactions in ZF5 than ZLD. Together, these data reveal that the differential activity of *Antp* is mediated by an increase in the duration and frequency of ZF5 hub-locus interactions.

### Zelda’s target selectivity is determined by cofactor interactions when its DBD is perturbed

**Fig. 6:**
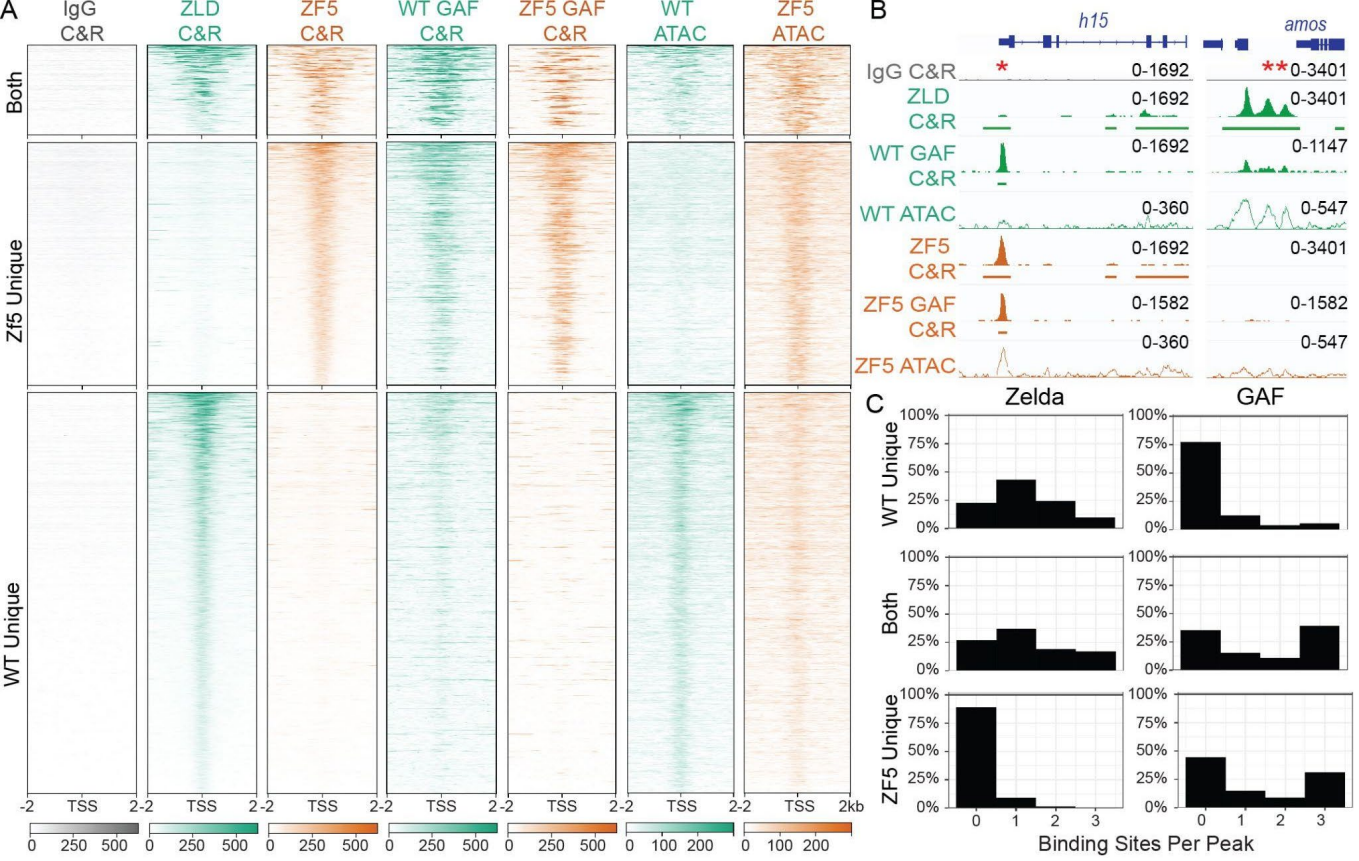
Zelda DNA binding mutant relocates to sites of GAF binding. **(A)** Heatmap of Zelda peaks at TSSs separated by whether they are present in WT only (2268), ZF5 only (1478), or both (653). The heatmaps display ZLD and ZF5 CUT&RUN (C&R), GAF CUT&RUN in WT and ZF5 embryos, and ATAC-seq in WT and ZF5 embryos. **(B)** Example ZLD, ZF5, and GAF tracks (Solid), ATAC-seq (hollow), and called peaks (solid bars). *denotes a GAF peak where ZF5 is relocalized to. **denotes a ZLD peak that is lost in ZF5. **(C)** Histograms of binding site counts in ZF5s unique binding sites, WT Zelda unique binding sites, and binding sites shared by both. Hits must match the consensus sequence by 90% for Zelda, and 85% for GAF.

Although Zelda is a key activator of the zygotic genome (26) and a primary driver of accessibility in the early embryo (24, 25), it has been shown that sites bound by another pioneer factor GAGA-factor (GAF) remain accessible in Zelda-null embryos (31), and through individual depletions of GAF or ZLD, that their binding is independent of each other (40). Furthermore, in the absence of Zelda, the morphogenic TF Dorsal has been shown to relocate to sites that are enriched for GAF motifs but depleted in ZLD motifs (41). Strikingly, in larval brains where Zelda’s canonical motif alone cannot explain its binding preferences, Zelda peaks are more likely to be found at GAF motifs (42). We hypothesized that the relocalization of ZF5 is driven by interactions with co-factors recruited to sites that are pioneered and occupied by GAF. To test this idea, we compared GAF binding in wildtype and ZF5 embryos using CUT&RUN and found that GAF binding is highly correlated with sites that are differentially occupied by ZF5, and those that are occupied by both ZF5 and ZLD, but not at sites where ZF5 binding is lost (Fig. 6A–B). Consistent with previous reports on GAF binding in Zelda null embryos (40), GAF binding is not significantly altered in the ZF5 background (Fig. 6A and S11A). An examination of the motifs under ZLD and ZF5 peaks showed that while, as expected, Zelda motifs are enriched under ZLD peaks, in ZF5 this enrichment is lost and instead an enrichment of GAF motifs is found (Fig. 6C). These peaks also correspond to sites where Dorsal relocates upon Zelda depletion (41) (Fig. S11B) and are enriched in promoter regions relative to ZLD (Fig. S11C). These analyses suggest that ZF5s relocation to new target sites is largely driven by interactions with co-factors that are themselves redistributed when Zelda’s occupancy at its canonical targets is perturbed. Although this redistribution is correlated with GAF binding, differential transcription and accessibility is further enhanced by the recruitment of ZF5. This increased expression and accessibility indicate that Zelda’s disordered activation and interaction domains are acting functionally at these new sites when its hubs are relocalized there.

## DISCUSSION

The ability to measure molecular kinetics *in vivo* has revealed that most TFs bind chromatin with residence times on the order of just tens of seconds or less. Instead of the duration of binding events tuning TF target site occupancy, it is determined by their frequency, which can be increased by accumulating TFs in the vicinity of their target sites. These local accumulations represent distinct nuclear microenvironments (4) which are in many cases driven by low-affinity but selective interactions between the IDRs of TFs (14, 15). At super-enhancers such microenvironments (composed of Mediator and RNA Pol II) are more stable in time and exhibit liquid-like droplet properties, and are as such referred to as condensates (9, 12, 13). However, persistent condensate-like bodies that persist for minutes or longer only represent ~10% of the observed clusters in these studies, with the global average lifetime of Mediator and RNA Pol II clusters only lasting ~11 seconds and ~13 seconds respectively (12, 43), consistent with transient TF hubs in *Drosophila* embryos. The challenges involved in characterizing transient TF hubs at endogenous concentrations in *in vivo* contexts has hindered our ability to understand how these hubs are targeted to specific gene targets and the molecular kinetics underlying their formation.

**Fig. 7:**
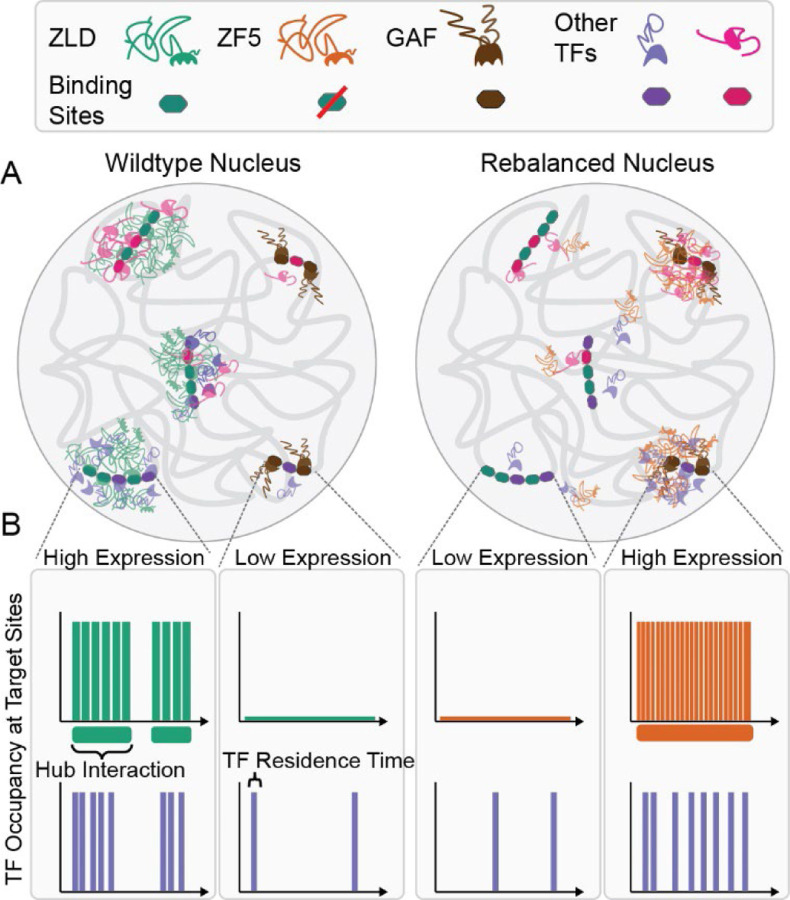
A model for hub relocalization due to a kinetic rebalancing in the nucleus. **(A)** In nuclei from wildtype embryos (left) Zelda binds to its strong DNA motifs, pioneers them and drives transcription by both enabling the binding of and recruiting co-factors and additional TFs in locally enriched hubs. In nuclei from ZF5 embryos (right), Zelda no longer binds its cognate motifs, it and its cofactors are now drawn to orthogonal sites where they form new types of hubs, many of which are bound by GAF. **(B)** Although the residence time of ZF5 on chromatin is severely reduced, its hubs are now more stable in time and interact more frequently with new target sites, where they drive high occupancy and expression.

Our initial observations that inhibiting the ability of Zelda to recognize its cognate motifs functionally relocalizes it to new target sites (Figs. 1 and 2) opened the opportunity to investigate the mechanisms of hub targeting in the endogenous context of live developing embryos. The fact that ZF5 mutant Zelda relocalizes to GAF sites along with TFs that normally exhibit Zelda-dependent binding leads us to a model in which hub targeting is dictated by the relative strengths and abundances of co-binding factors and protein-chromatin interactions (Fig. 7). Although the strong binding of wildtype Zelda to its canonical target sites is likely less sensitive to the presence of cofactors given its pioneering function, as was recently described for the yeast transcription factor Msn2 (44), the relocalization of the ZF5 mutant to sites occupied by GAF enforces the idea that site selectivity for most TFs is determined by both their DBD activity and interactions with co-binding factors through non-DBD regions. Many TFs binding sites are low-affinity in nature, and thus depend on the presence of co-binding factors to occupy their sites (45). Indeed, locally high-concentrations of TFs, such as those found in hubs, can render low-affinity binding sites to functional (46). When Zelda can no longer bind its cognate sites, the TFs which co-occupy these sites with Zelda relocalize to their next highest affinity available targets. Our data suggest that ZF5 retains the ability to interact with these co-binding partner TFs and thus relocalizes along with them. The formation and localization of hubs thus represents a complex kinetic balancing act, involving transient low-affinity interactions that determine the recruitment of different transcription factor families to target sites.

Transcription factors, including pioneer factors, play differential roles throughout development and in different tissue contexts, and often exhibit context specific site selection based on the presence of cofactors and their binding motifs at these sites (47–50). For example, beyond its role in embryos, Zelda is expressed in larval brains where it promotes neural stem cell fates by occupying a different subset of target sites with no distinct DNA sequence features other than a high correlation with GAF binding (42). Given Zelda’s role in mediating the binding of a large majority of developmental TFs to their targets in embryos (24), our results suggest that the occupation of a site by a given TFs is orchestrated by the recruitment of likely compositionally distinct hubs, dictated by the underlying genomic sequence which drives the presence of a multitude of binding partners.

Our observation that Zelda functionally relocalizes to sites where there is no DNA binding motif present (Fig. 6C) also forces us to further reconsider how we define functional binding sites for TFs. For example, in many previous single-molecule studies, the deletion or mutation of a TFs DBD, which has resulted in the loss of the longer-lived population in survival probability curves as we have observed for ZF5 (Fig. 2), has been interpreted as a loss in specific binding and often used to calculate parameters such as search time (7, 33, 34, 51, 52). Furthermore, recent domain mapping single-molecule tracking experiments on the histone acetyltransferase P300 have also highlighted that individual interaction domains of TFs, beyond their DBD, are also insufficient to explain targeted recruitment (53). Our results further emphasize the need to think of target selectivity and specificity not from the perspective of just binding motifs but from the perspective of the family of factors recruited to each individual site.

Although it is recognized that multifactor TF condensates and hubs likely mediate the occupancy of such families of factors, they are often viewed as having consistent emergent properties depending on the primary, or scaffold protein driving their formation. This view that key properties such as lifetimes, relative enrichments, and sizes are singular, is based on an assumption of the role of liquid-liquid phase separation in driving their formation (54). Our finding that the lifetimes of individual Zelda hubs vary considerably within a single nucleus suggests that their stability is tuned in a target-specific manner (Fig. 4). The increase in hub lifetimes at ectopic sites in the ZF5 mutant leads us to speculate that specific co-binding factor interactions are key to tuning the stability and frequency of hub interactions. Our results also highlight potential variability in the functional roles of hubs; for example, hub formation at one target may indeed lead to a loss of occupation at other sites leading to lower gene expression as has been observed in the case where hub function is assessed at an individual site (55).

Our observation that the residence times of the ZF5 mutant Zelda are reduced almost five-fold over the wildtype protein while it forms longer lived hubs (Fig. 4) that interact more frequently with a target site (Fig. 5) leads us to a model in which hub stabilities and proximities are key determinants driving TF occupancy at a target (Fig. 7). We speculate that underlying combinations of low-affinity binding sites, presence and levels of co-binding factors, epigenetic modifications (56), and local chromatin organization together tune hub stabilities and interaction frequencies. We posit that these local properties have been optimized over the course of evolution to tune TF occupancy at specific loci depending on cellular context. In situations where this fine balance has been disrupted, hubs might be aberrantly stabilized leading to disease, as has been observed in the case of the chromatin reader protein ENL (57). The selection and occupation of gene targets by transcription factors that are determined by mechanisms outside of specific DNA motif recognition may carry unique advantages at evolutionary timescales as they don’t require changing or evolving new DNA binding motifs. We further speculate that over the course of evolution such weak but tunable interactions, along with the plastic nature of IDRs, have allowed for maximal changes in phenotype, with minimal perturbations to overall topology of the highly pleiotropic gene regulatory networks that are a hallmark of early development.

While our results here deepen our understanding of the fine balance of interaction kinetics that drive TF hub targeting, further exploration is needed to elucidate the rules that dictate essential properties such as hub composition and formation probabilities. Interdisciplinary approaches such as we have developed here using a combination of high-resolution volumetric imaging to track hub properties at target loci, single-molecule tracking to understand the impact of hub formation on molecular interactions, and genomics to assess functional effects, will be critical to tackling these essential questions on the fundamentals of gene regulation.

## Supplementary Material

Supplement 1

Supplement 2

Supplement 3

Supplement 4

Supplement 5

Supplement 6

Supplement 7

Supplement 8

Supplement 9

Supplement 10

Supplement 11

Supplement 12

Supplement 13

Supplement 14

Supplement 15

Supplement 16
