# Supplementary material for: A fine kinetic balance of interactions directs transcription factor hubs to genes
Source: bioRxiv. 2024 Oct 15:2024.04.16.589811. Originally published 2024 Apr 16. Preprint. [Version 3] doi: 10.1101/2024.04.16.589811 (PMC11042322; doi:10.1101/2024.04.16.589811)
Supplement: Supplement 1 [file media-1.pdf]

Supplementary Materials for:

Samantha Fallacaro[1,2,3]†, Apratim Mukherjee[1,2]†, Puttachai Ratchasanmuang[2,4]†, Joseph Zinski[1,2]†, Yara I Haloush[2], Kareena Shankta[2,5], Mustafa Mir[1,2,4,6]*

[1]Department of Cell and Developmental Biology, Perelman School of Medicine, University of Pennsylvania; Philadelphia, PA 19104, USA.
[2]Center for Computational and Genomic Medicine, Children's Hospital of Philadelphia; Philadelphia, PA 19104, USA.
[3]Developmental, Stem Cell, and Regenerative Biology Graduate Group, Perelman School of Medicine; Philadelphia, PA 19104, USA.
[4]Howard Hughes Medical Institute, Children's Hospital of Philadelphia; Philadelphia, PA 19104, USA.
[5]Roy and Diana Vagelos Program in Life Sciences and Management, University of Pennsylvania; Philadelphia, PA 19104, USA.
[6]Epigenetics Institute, University of Pennsylvania Perelman School of Medicine; Philadelphia, PA 19104, USA.
†These authors contributed equally to this work. *Correspondence to: Mustafa Mir Email: mirm@chop.edu

List of Supplementary Materials

Fig. S1: Creation of mutant Zelda fly lines.
Fig. S2: Characterization of hubs
Fig. S3: CUT&RUN and RNA-seq in ZLD and ZF5 embryos
Fig. S4: Differential chromatin accessibility
Fig. S5: Single molecule tracking data analysis
Fig. S6: Analysis pipeline for Zelda hub stability across mutants
Fig. S7: Hub lifetimes quantified across Zelda mutants.
Fig. S8: ZF5 accumulation at Antp.
Fig. S9: Zelda mutant relocalization.

Movie 1. Volumetric Imaging of all Zelda mutants in nc13.
Movie 2. Single-Molecule Imaging for determining residence times.
Movie 3. Single-Molecule Imaging for quantifying diffusion kinetics of mEos3.2-ZLD.
Movie 4. Single-Molecule Imaging for quantifying diffusion kinetics of mEos3.2-ZF5.
Movie 5. Volumetric Imaging of ZLD and ZF5 in nc14.
Movie 6. Volumetric Imaging of ZLD.
Movie 7. Volumetric Imaging of ZF5.
Movie 8. Volumetric Imaging of sfGFP-ZLD.
Movie 9. Hub Segmentation.
Movie 10. Volumetric Imaging of ZF5/+.
Movie 11. Volumetric Imaging of ΔIDR/+.
Movie 12. Volumetric Imaging of ZF1-6/+.
Movie 13. Simultaneous volumetric Imaging of ZLD in the context of nascent transcription of Antp.
Movie 14. Simultaneous volumetric Imaging of ZF5 in the context of nascent transcription of Antp in nc12.
Movie 15. Simultaneous volumetric Imaging of ZF5 in the context of nascent transcription of Antp in nc13.
Movie 16. Simultaneous volumetric Imaging of ZF5 in the context of nascent transcription of Antp in nc14.

Table S1. Summary of RNA-seq experiments and associated file names submitted to GEO
Table S2. Summary of CUT&RUN and ATAC-seq experiments and associated file names submitted to GEO
Table S3. Summary statistics of differentially expressed genes (download .csv from supplementary materials)

Table S4. Summary statistics for single-molecule data.
Table S5. Summary statistics of hub properties from Fig. 1 and 4
Table S6. Summary statistics of hub lifetimes from Fig. 4
Table S7. Summary statistics of hub-target gene interactions from Fig. 5

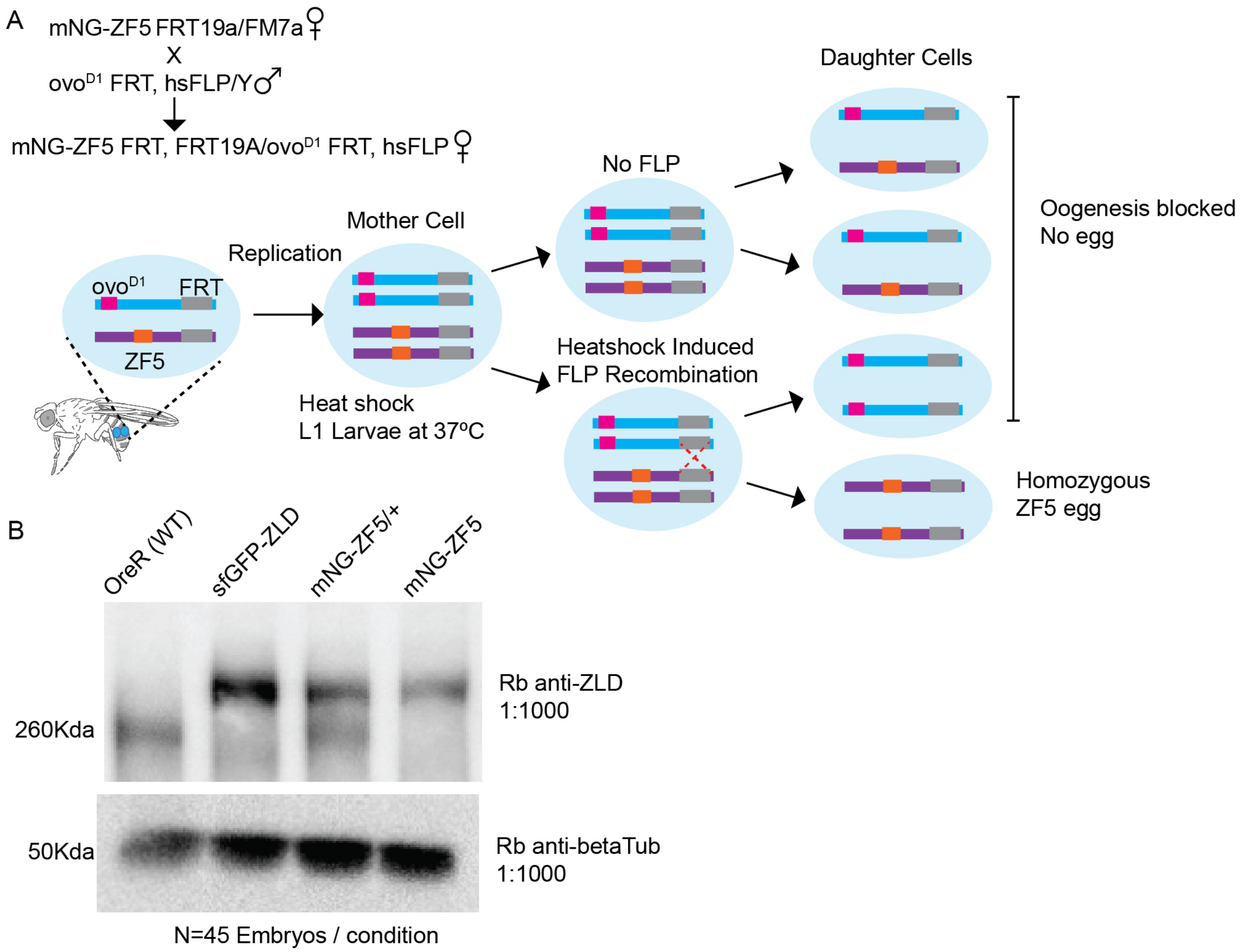

Fig S1: Creation of mutant Zelda fly lines. (A) Scheme for generation of germline clones containing homozygous mutations for Zelda using the FLP/FRT system (figure adapted from https://bdsc.indiana.edu/stocks/recombinases/dfs.html). **(B)** Western blots to assess if any wild type Zelda protein is present in embryos generated from germline clones.

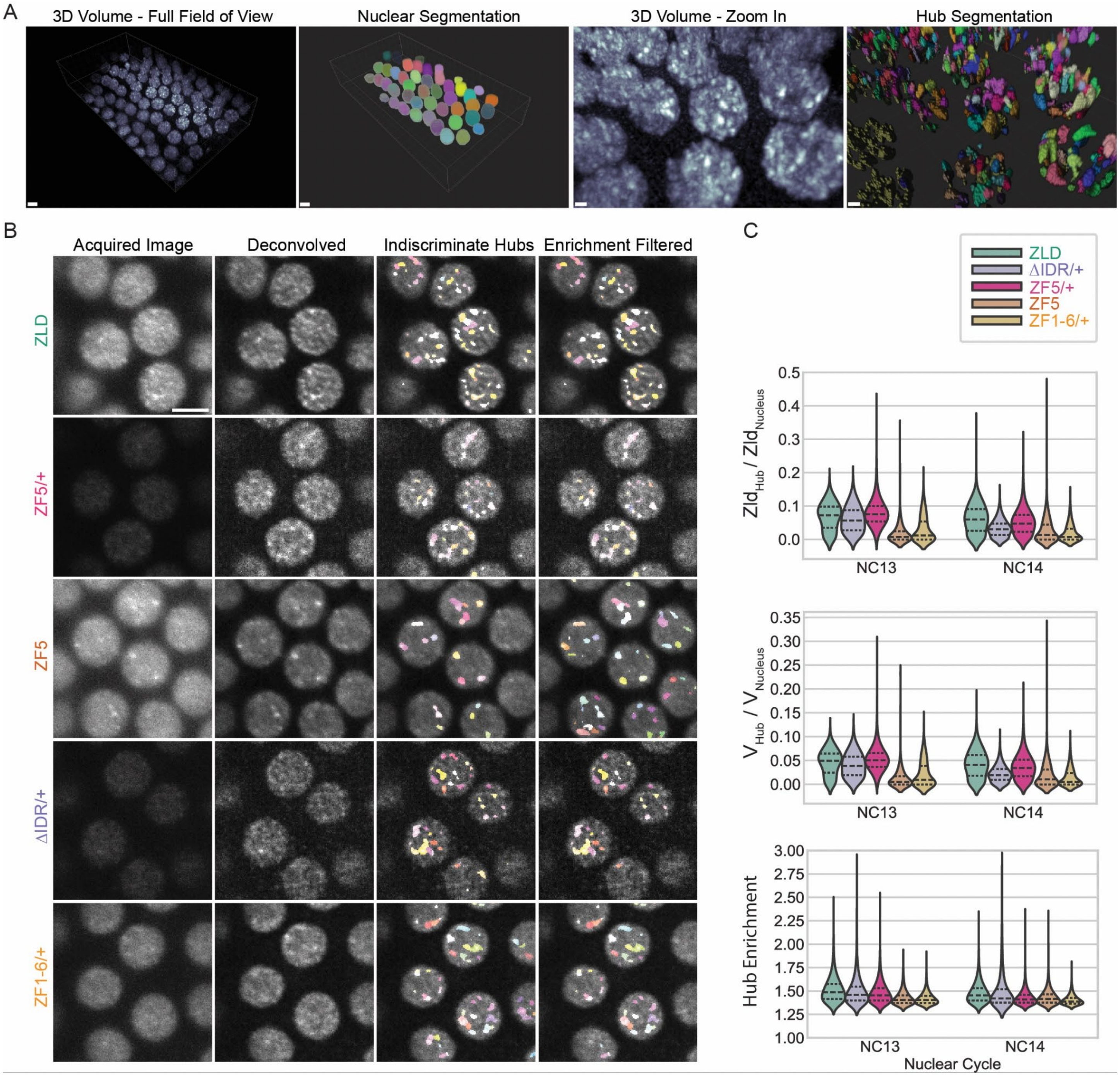

Fig S2: Characterization of Zelda hubs. (A) Representation of hub analysis: volumes of a layer of nuclei on the *Drosophila* embryo surface are acquired during imaging and deconvolved (left). Scale bar = 5 µm. Nuclei are segmented in 3D using a trained machine learning model (middle left). Scale bar is 5 µm. Zelda shows non-uniform distributions within single nuclei (middle right). Scale bar = 1 µm. Hub segmentation is performed in 3D using a custom analysis pipeline (right). Scale bar = 1 µm. **(B)** High contrast imaging of mNeonGreen tagged Zelda embryos (ZLD, ZF5/+, ZF5, ΔIDR/+, or ZF1-6/+) in the middle of nc13 before and custom hub segmentation pipeline outputs. Images shown are from a single z-slice in each volume. Acquired images column is shown with consistent contrast adjustment to assess expression levels, while the second column is shown with individual contrast limits adjusted to better visualize hubs. Third column shows initial segmentation output where hubs are indiscriminately called, and third column is after filtering when only hubs with enrichment scores

at 0.5 cumulative probability of the wildtype ZLD are considered. Scale bar = 5 µm. **(C)** Quantification of biophysical hub properties including proportion of Zelda within hubs (sum intensity) compared to all nuclear Zelda (sum intensity), the volume fraction of hubs within the nucleus, and hub enrichment (mean hub intensity normalized using mean nuclear intensity) from a snapshots taking in the middle of interphase during nc 13 and 14. N-values and statistics are summarized in Table S5.

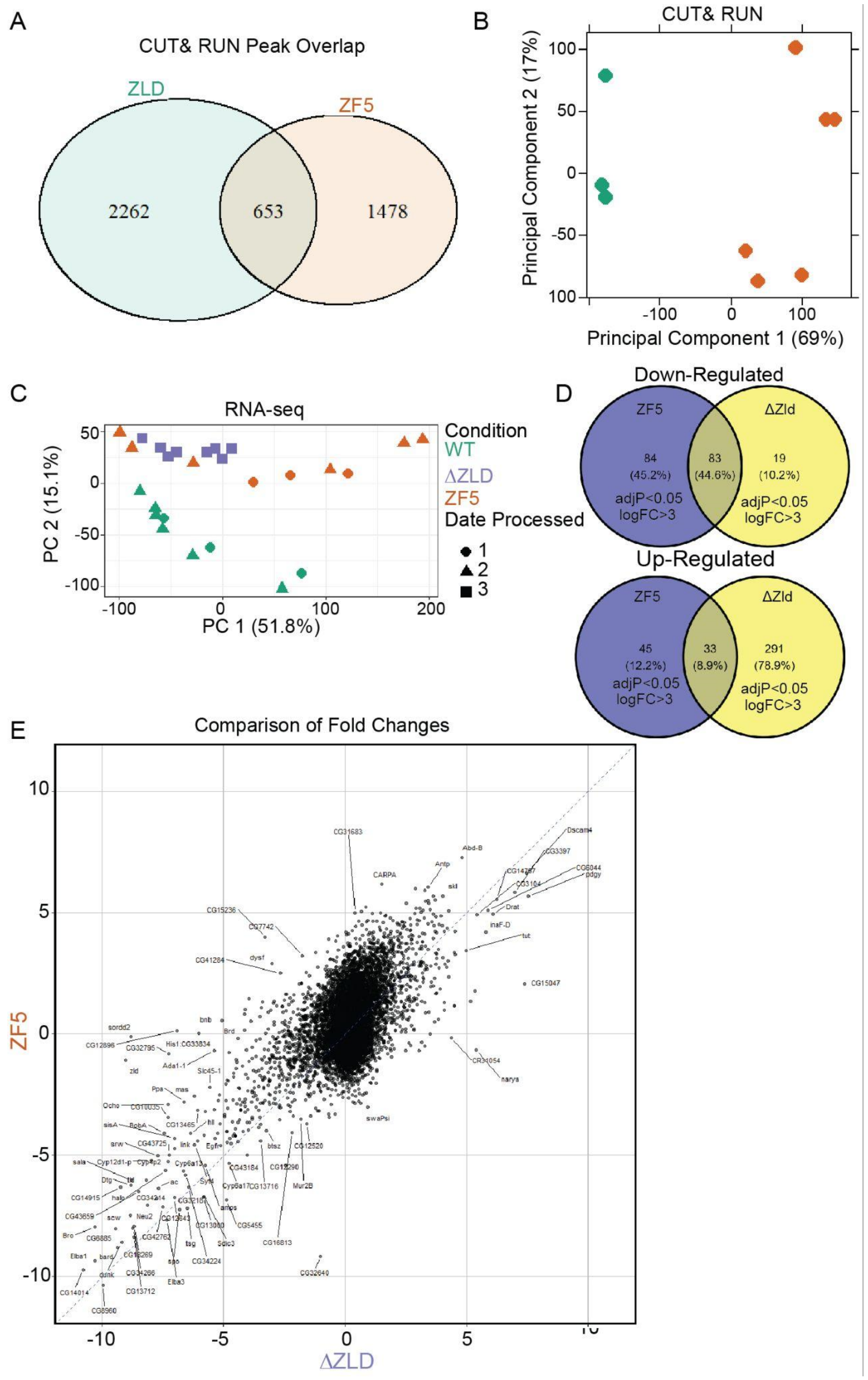

Fig. S3. CUT&RUN and RNA-seq (A) A comparison of overlap in identified peaks in CUT&RUN experiments on Wildtype and ZF5 embryos. **(B)** Principal component analysis of replicates from which peaks were calculated in (A). **(C)** Principal component analysis on RNA-seq data. **(D)** Overlap of differentially regulated genes in ZF5, and Zelda knockout embryos **(E)** Scatter plot of all ZF5 vs. Zelda knockout RNA-seq gene expression data.

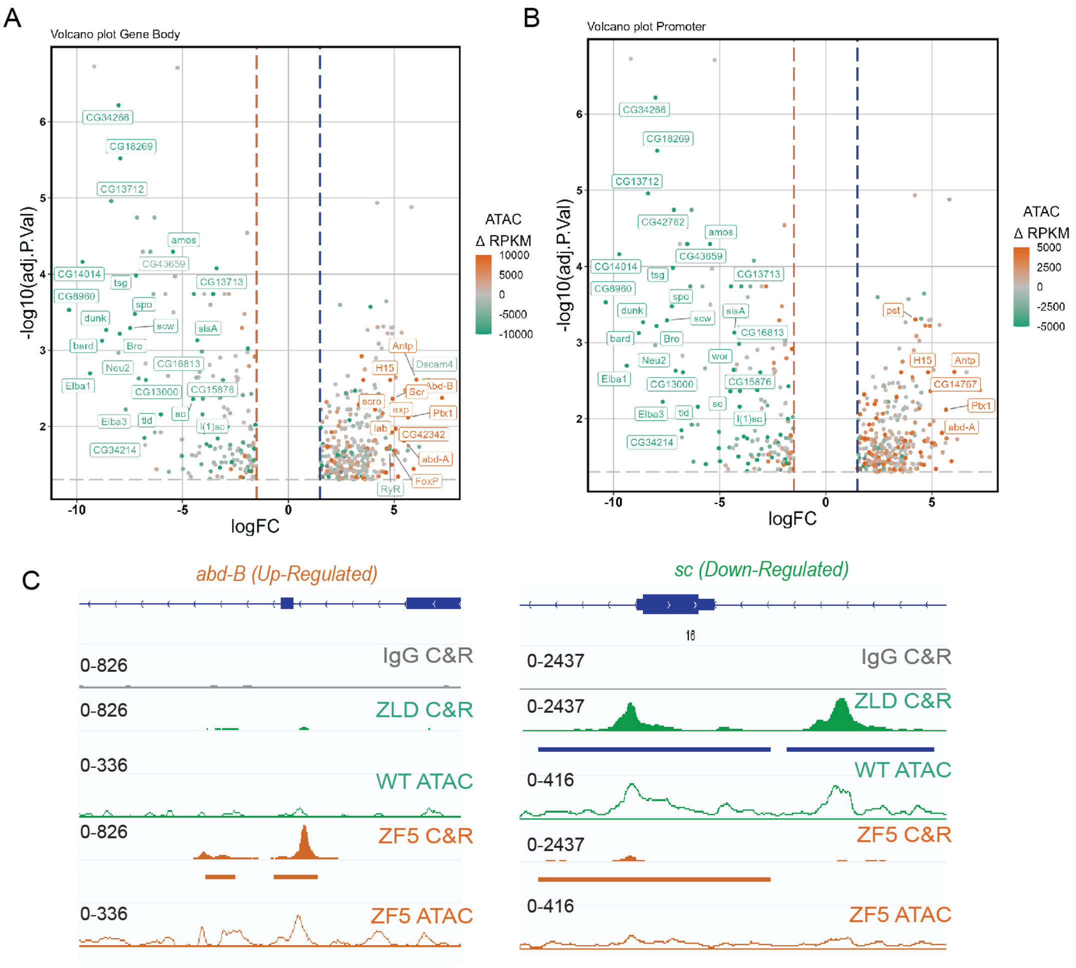

Fig. S4. Changes in accessibility at up- and down-regulated genes (A) Volcano plots of genes up- and down-regulated in ZF5 mutant measured via RNA-seq. Genes with a significance <0.01 and Log Fold Change > 4 and a difference in RPKM > 5000 (from CUT&RUN) are labeled . The gray dotted line marks adjust P-value = 0.05, blue and red dotted lines are +/-1.5 LCF. Points are colored by the difference in Zelda RPKM between WT and ZF5 mutants across the gene body of each gene. **(B)** Points are colored by the difference in ATAC RPKM between WT and ZF5 mutants within 2kb of the TSS. **(C)** Example ZLD and ZF5 CUT&RUN tracks (solid), ATAC (hollow), and called peaks (solid bars below tracks) of up- and down-regulated genes.

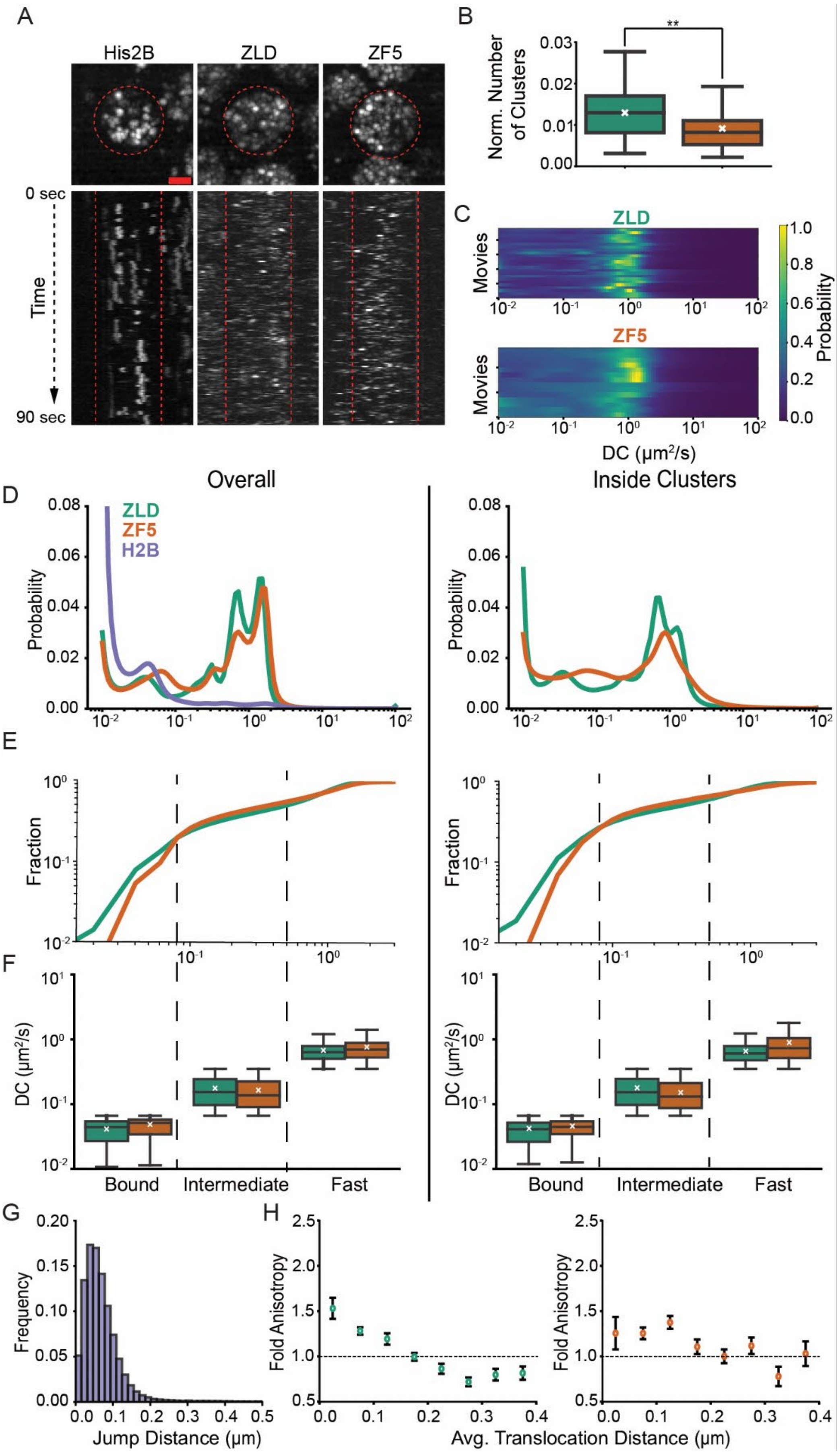

A
His2B
ZLD
ZF5
0 sec
Time
90 sec
B
Norm. Number of Clusters
**
C
ZLD
ZF5
Movies
Probability
DC (μm²/s)
D
Overall
Inside Clusters
ZLD
ZF5
H2B
Probability
E
Fraction
F
DC (μm²/s)
Bound
Intermediate
Fast
G
Frequency
Jump Distance (μm)
H
Fold Anisotropy
Avg. Translocation Distance (μm)

Fig. S5. Single molecule tracking data analysis. **(A)** Maximum intensity projections over 90 seconds of raw single molecule tracking data. Red circles indicate nuclear boundaries. Kymographs (x-t maximum projections) of single molecule detections of mEos3.2-ZLD, mEos3.2-ZF5, and H2B-mEos3.2. Acquired with an exposure time of 500 msec, scale bar is 2 μm. **(B)** Quantification of cluster numbers normalized by the total trajectories per nucleus for ZLD (n=172) and ZF5. (n=84). **(C)** Heatmaps of the probability spectra of diffusion coefficients obtained for each movie (one movie/row). Data was analyzed over 23 movies from 7 embryos for ZLD and 14 movies from 3 embryos for ZF5. **(D)** Diffusion coefficient spectra for ZLD, ZF5 and H2B for all trajectories (left) and for ZLD and ZF5 trajectories inside clusters (right). A total of 70,827, 40,307, and 51,158 tracks were measured for ZLD, ZF5 and H2B cases respectively overall, and 14,516 tracks and 3,375 tracks were measured for ZLD and ZF5 respectively in clusters **(E)** Cumulative distribution of diffusion coefficients (DC) for ZLD and ZF5 overall (left) and inside clusters (right). Black dashed lines are cutoffs for bound, intermediate, and fast trajectories. **(F)** Quantification of the average diffusion coefficient in each kinetic bin for ZLD and ZF5 overall (left) and inside clusters (right). White cross is the mean, error bars represent standard deviations from bootstrapping analysis. **(G)** Histogram of jump distances from H2B trajectories used to determine the distance threshold for anisotropy analysis. **(H)** Fold anisotropy as a function of average translocation distance (over two consecutive jumps) for ZLD (left) and ZF5 (right) overall. Error bars represent standard deviation from bootstrapping analysis. Dashed line indicates an isotropic distribution.

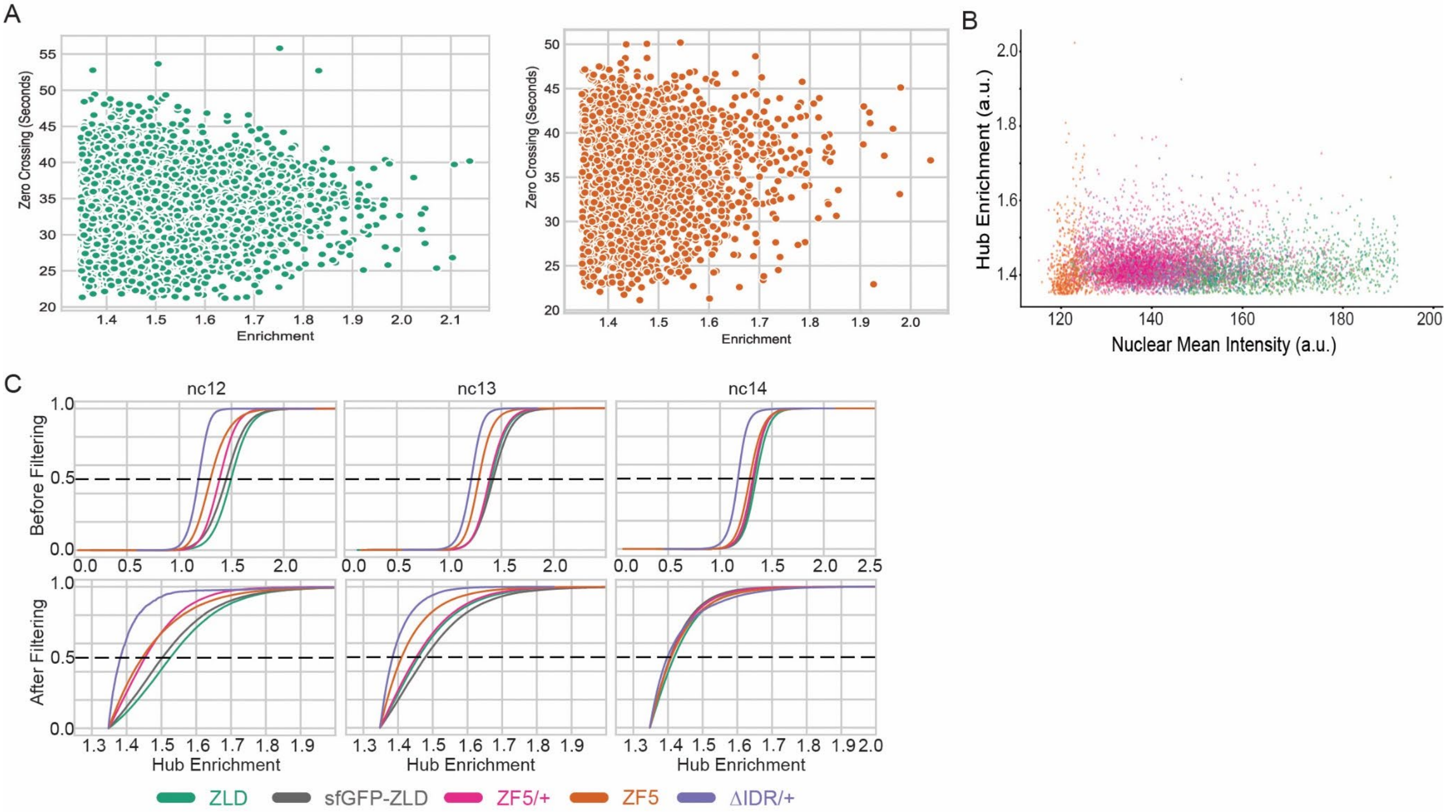

Fig. S6: Analysis of Zelda hubs across mutants. (A) Quantification of hub enrichment along with the zero crossing in seconds representing the hub lifetimes show no correlation in either ZLD (top) or ZF5 (bottom) between enrichment and lifetime of each hub. **(B)** Scatter plot showing hub enrichment as a function of nuclear mean intensity. Hub enrichment quantification is not impacted by signal-to-noise difference among mutants. **(C)** Cumulative distributions of hub enrichment for each Zelda mutant before and after filtering (top and bottom respectively).

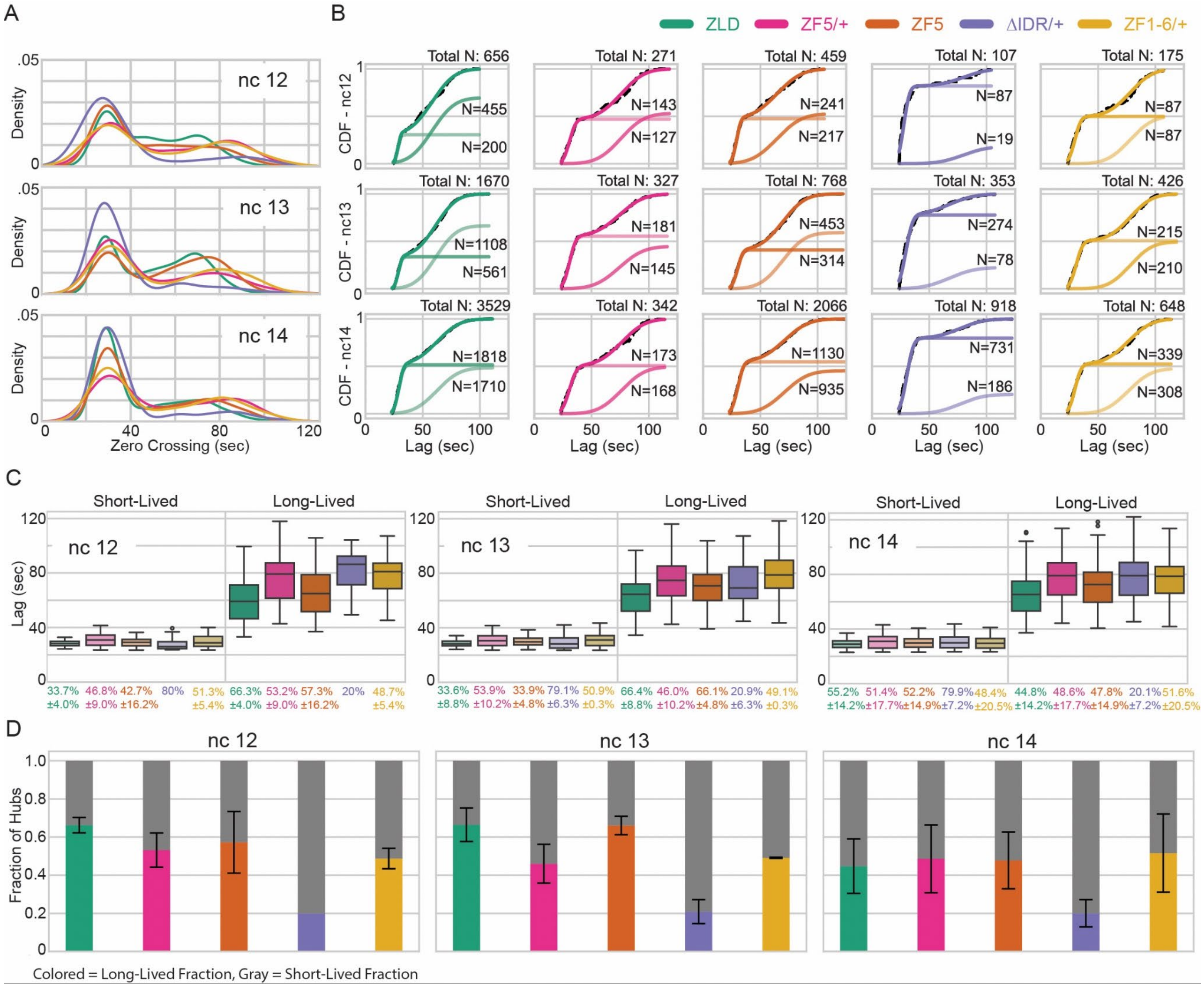

Fig. S7: Hub lifetimes quantified across Zelda mutants. (A) Density plots for Zelda mutants showing zero crossing of the autocorrelation of intensity of a 1 μm^3 box centered at each hub. Distributions are not normal but suggest a two-component mixture of hubs. **(B)** Cumulative distributions of zero crossings from original data (black dotted line), fitted CDF (solid dark color), and two gaussian components (lighter colors). N values provided for the number of hubs fit into these components. For nc12, total N is derived from 3 ZLD, 4 ZF5/+, 5 ZF5, 2 ΔIDR/+, and 3 ZF1-6/+ embryos. For nc13, total N is derived from 6 ZLD, 3 ZF5/+, 3 ZF5, 3 ΔIDR/+, and 3 ZF1-6/+ embryos. For nc14, total N is derived from 9 ZLD, 3 ZF5/+, 9 ZF5, 4 ΔIDR/+, and 4 ZF1-6/+ embryos. **(C)** Zero-crossing distributions shown in box-plots from each component (short- and long-lived) for nc12, nc13, and nc14 for Zelda mutants. Proportion of hubs within each of the conditions is as written underneath each box plot **(D)** Proportion of hubs in each life-time category. A Kruskal-Wallis test followed by Mann-Whitney U Test was performed among all pairings in the same component in the same nc. All mutants are significantly different from ZLD. Other pairings and all statistical analyses are summarized in Table S6.

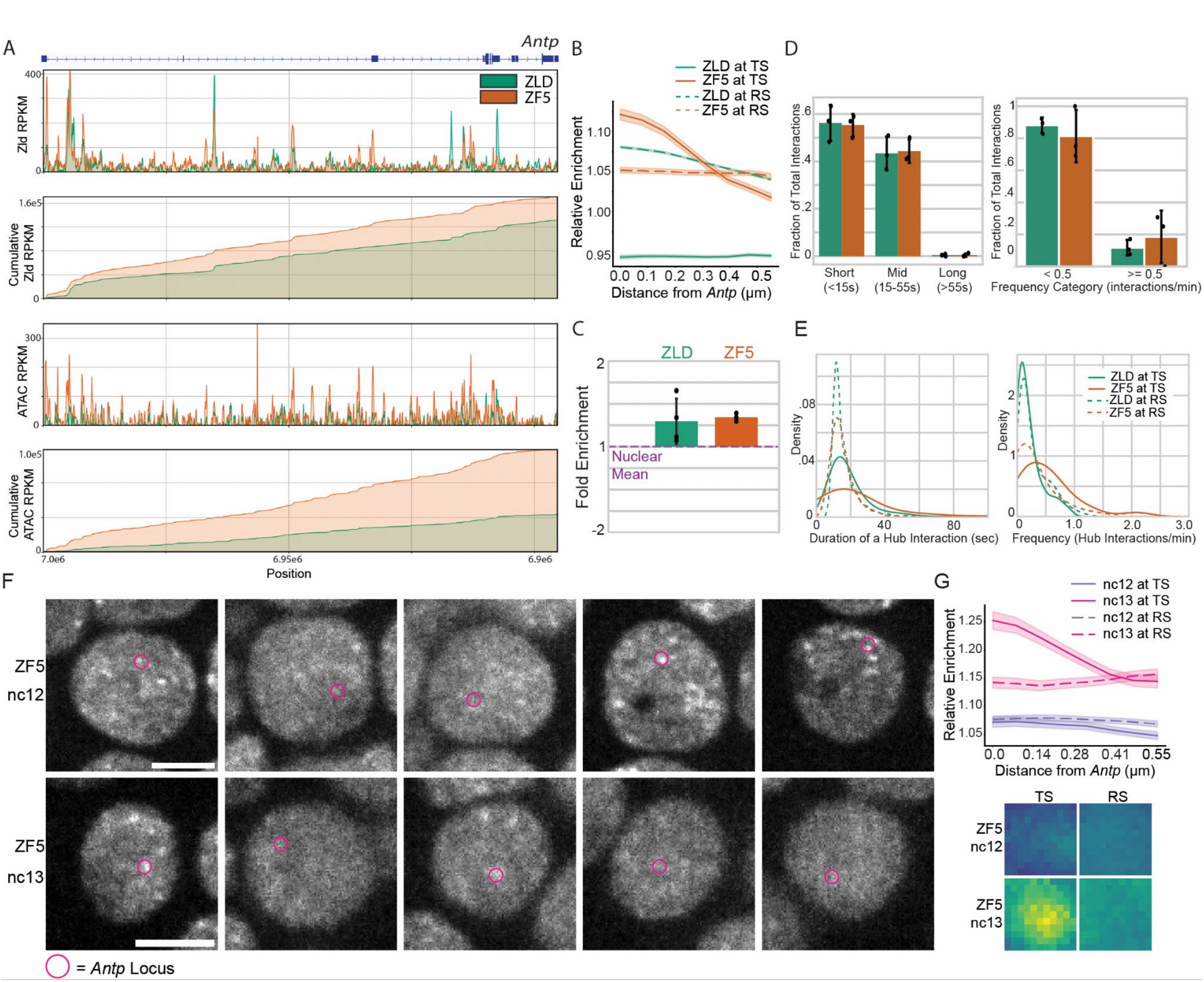

Fig. S8. ZF5 accumulation at *Antp*. **(A)** Measured and Cumulative RPKM from CUT&RUN against ZLD and GAF, and ATAC-seq, in WT and ZF5 embryos, along the *AntP* locus. **(B)** Average radial profile with standard error centered at the *Antp* shows ZLD or ZF5 enrichment as a function of distance from MS2 spot center (or transcription site (TS)) or at a control spot randomly placed in the nucleus (or random spot (RS)). **(C)** Fold enrichment of mNeonGreen intensity above the nuclear mean intensity within a 0.55 µm radius centered at a random spot within ZLD and ZF5 nuclei (n=3 embryos for ZLD and n=3 embryos for ZF5, more statistics summarized in Table S7). **(D)** Fractions of hub interactions per embryo of duration of individual hub interactions (left) and the frequency of interactions (right) of a random spot within ZLD and ZF5 nuclei in nc14. **(E)** Distribution of the frequency and duration of hub interactions at *Antp* locus (TS) or random spot (RS) (n=172 nuclei for ZLD and n=65 nuclei for ZF5, more statistics summarized in Table S7). **(F)** Representative images (single slice in Z) of five different nuclei for ZLD and ZF5. Zelda is shown in grayscale while the location of the MCP-mCherry at *Antp-MS2* locus is shown as a pink circle on the images. Scale bar is 5 µm. **(G)** Cropped images show average intensity centered either at the transcription site (TS) or random site (RS) in the nucleus. Image size is 1.1 µm x 1.1 µm. All cropped images have the same contrast adjustment. Average radial profile with standard error centered at the *Antp* shows Zelda ZF5 in either nc12 (purple) or nc13 (pink) enrichment as a function of distance from MS2 spot center. Two embryos were analyzed in nc12 and one in nc 13.

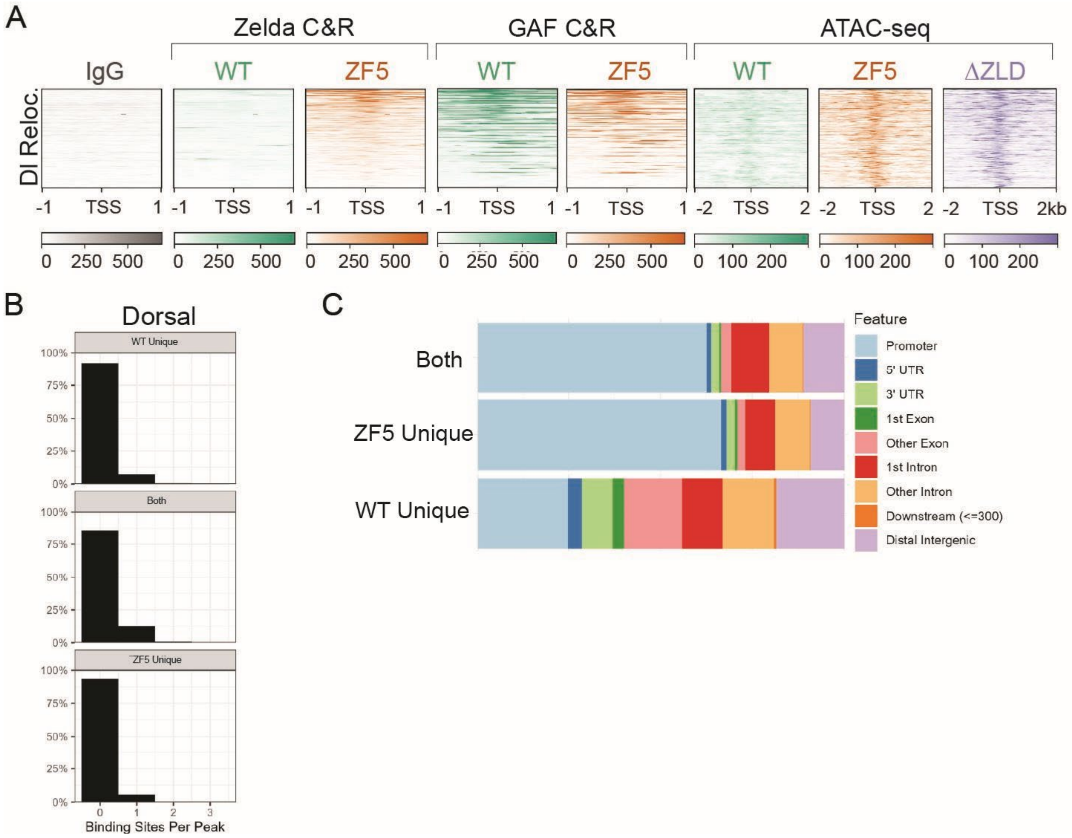

Fig. S9: Zelda DNA binding mutant relocalization. (A) ZLD and GAF CUT&RUN (C&R), and ATAC-seq in WT and ZF5 at sites of Dorsal relocalization in ΔZLD null embryos as identified in Sun et al. 2015[43]. **(B)** Histograms of Dorsal binding motif counts in ZF5s unique, WT Zelda unique, and shared binding sites. **(C)** Annotation of peak call locations.

Table S1. Summary of RNA-seq experiments and associated file names submitted to GEO

Sample/Filename	Condition	Day	Genotype	Read Count
H2B_GFPwt1_1_1	WT	2	H2B-eGFP (III)	21044634
H2B_GFPwt2_2_1	WT	2	H2B-eGFP (III)	17390264
H2B_GFPwt2_3_1	WT	2	H2B-eGFP (III)	17693844
H2B_GFPwt2_5_1	WT	2	H2B-eGFP (III)	23905163
H2B_GFPwt3_1	WT	2	H2B-eGFP (III)	16472166
H2B_GFPwt4_1	WT	2	H2B-eGFP (III)	21418487
znf5mtGLC2_1_1	Zf5	2	mNGzld_znf5mt_FRT19A/mNGzld_znf5mt_FRT19A	13801043
znf5mtGLC2_6_1	Zf5	2	mNGzld_znf5mt_FRT19A/mNGzld_znf5mt_FRT19A	20463584
znf5mtGLC2_8_1	Zf5	2	mNGzld_znf5mt_FRT19A/mNGzld_znf5mt_FRT19A	13055948
znf5mtGLC2_9_1	Zf5	2	mNGzld_znf5mt_FRT19A/mNGzld_znf5mt_FRT19A	15760098
znf5mtGLC2_12_1	Zf5	2	mNGzld_znf5mt_FRT19A/mNGzld_znf5mt_FRT19A	29243336
znf5mtGLC2_13_1	Zf5	1	mNGzld_znf5mt_FRT19A/mNGzld_znf5mt_FRT19A	20783123
mNGznf5_GLC1_1	Zf5	1	mNGzld_znf5mt_FRT19A/mNGzld_znf5mt_FRT19A	19518017
mNGznf5_GLC	Zf5	1	mNGzld_znf5mt_FRT19A/mNGzld_znf5mt_	18432907

2_1			FRT19A	
mNGznf5_GLC3_1	Zf5	1	mNGzld_znf5mt_FRT19A/mNGzld_znf5mt_FRT19A	23425471
sfGFPzldwt_1B_1	WT	1	sfGFPzld-wt FD1	21583776
sfGFPzldwt_3_1	WT	1	sfGFPzld-wt FD1	13681957
sfGFPzldwt_5B_1	WT	1	sfGFPzld-wt FD1	23554319
Zld294_GLC_1_1	Zld294	3	Zld294_FRT19A/Zld294_FRT19A	20494620
Zld294_GLC_2_1	Zld294	3	Zld294_FRT19A/Zld294_FRT19A	21489357
Zld294_GLC_3_1	Zld294	3	Zld294_FRT19A/Zld294_FRT19A	21032008
Zld294_GLC_4_1	Zld294	3	Zld294_FRT19A/Zld294_FRT19A	19203330
Zld294_GLC_5_1	Zld294	3	Zld294_FRT19A/Zld294_FRT19A	21287685
Zld294_GLC_6_1	Zld294	3	Zld294_FRT19A/Zld294_FRT19A	19614810
Zld294_GLC_7_1	Zld294	3	Zld294_FRT19A/Zld294_FRT19A	21877546
Zld294_GLC_8_1	Zld294	3	Zld294_FRT19A/Zld294_FRT19A	20532415

Table S2. Summary of CUT&RUN and ATAC-seq experiments and associated file names submitted to GEO

Filename	Condition	Antibody	Assay	Date	Total Reads	% Aligned w/Bowtie2	Aligned Reads	RiP
ATAC_H2B	WT	NA	ATAC	230719	28344155	87.88	24908844	0.29
ATAC_Znf5_1	ZF5	NA	ATAC	230719	29298587	84.07	24631323	0.35
ATAC_Znf5_2	ZF5	NA	ATAC	230719	30503525	81.33	24808517	0.31
ATAC_Zld294_1	Zld294	NA	ATAC	240718	10205428	89	9082831	0.08
ATAC_Zld294_2	Zld294	NA	ATAC	240718	13155206	83.44	10976704	0.06
ATAC_Zld294_3	Zld294	NA	ATAC	240718	14384544	84.85	12205286	0.07
ATAC_Zld294_4	Zld294	NA	ATAC	240718	9220031	84.74	7813055	0.09
ATAC_Zld294_5	Zld294	NA	ATAC	240718	10205262	92.73	9463340	0.05
ATAC_Zld294_6	Zld294	NA	ATAC	240718	15458598	90.44	13980757	0.07
sfGFPzldwt_Gaf1	WT	Gaf	C&R	240323	7955238	16.95	1348413	0.28
sfGFPzldwt_Gaf2	WT	Gaf	C&R	240323	11662950	14.26	1663137	0.29

sfGFPzldwt_Gaf3	WT	Gaf	C&R	240704	37695502	15.25	5748565	0.33
sfGFPzldwt_Gaf4	WT	Gaf	C&R	240704	21234142	16.58	3520621	0.35
sfGFPzldwt_Gaf5	WT	Gaf	C&R	240704	22932052	15.52	3559055	0.14
sfGFPzldwt_Gaf6	WT	Gaf	C&R	240704	25896729	15.52	4019173	0.15
GLC_mNGzld_Gaf_1	ZF5	Gaf	C&R	231207	15290910	81.98	12535489	0.23
GLC_mNGzld_Gaf_2	ZF5	Gaf	C&R	240704	15293781	69	10552709	0.15
GLC_mNGzld_Gaf_3	ZF5	Gaf	C&R	240704	16422981	30.18	4956456	0.12
GLC_mNGzld_Gaf_4	ZF5	Gaf	C&R	240704	23625405	28.97	6844280	0.10
GLC_IgG_1	ZF5	IgG	C&R	230620	14094365	90.45	12748354	0.04
GLC_IgG_2	ZF5	IgG	C&R	231207	17240038	55.5	9568222	0.04
sfGFPzldwt_zld3	WT	Zld	C&R	231207	18168704	86.64	15741366	0.07
sfGFPzldwt_zld4	WT	Zld	C&R	231207	22504068	94.11	21178579	0.14
sfGFPzldwt_zld5	WT	Zld	C&R	231207	22142523	95.7	21190395	0.15
mNGzld_GLC_1	ZF5	Zld	C&R	230620	192874	86.89	167588	0.18

					44		61	
mNGzld_GLC_2	ZF5	Zld	C&R	230620	24786487	91.14	22590405	0.08
mNGzld_GLC_3	ZF5	Zld	C&R	230719	18340650	93.03	17062307	0.08
mNGzld_GLC_4	ZF5	Zld	C&R	231207	19221901	73.22	14074276	0.08
mNGzld_GLC_5	ZF5	Zld	C&R	231207	13504968	49.79	6724124	0.19
mNGzld_GLC_6	ZF5	Zld	C&R	231207	12479679	47.82	5967783	0.21

Table S3. Summary statistics of differentially expressed genes (download .csv from supplementary materials)

Table S4. Summary statistics for single-molecule data from Fig. 3 (download .csv from supplementary materials)

Table S5. Summary statistics of hub properties from Fig. 1 and 4 (download .csv from supplementary materials)

Table S6. Summary statistics of hub lifetimes from Fig. 4 (download .csv from supplementary materials)

Table S7. Summary statistics of hub-target gene interactions from Fig. 5 (download .csv from supplementary materials)

Movie Captions

Movie 1. Volumetric Imaging of all Zelda mutants in nc13. 4D volumetric imaging data showing side-by-side comparison of mNeonGreen-ZLD, mNeonGreen-ZF5/+, mNeonGreen-ZF5, mNeonGreen-ΔIDR/+, and mNeonGreen-ZF1-6/+ during interphase of nc13. Scale bar = 5 μm. (Related to Fig. 1).

Movie 2. Single-Molecule Imaging for determining residence times. Single molecule data acquired at an exposure time of 500 milliseconds for mEos3.2-His2B, mEos3.2-ZLD, and mEos3.2-ZF5. (Related to Fig. 3).

Movie 3. Single-Molecule Imaging for quantifying diffusion kinetics of mEos3.2-ZLD. Single molecule data acquired at an exposure time of 10 milliseconds for mEos3.2-ZLD (Related to Fig. 3).

Movie 4. Single-Molecule Imaging for quantifying diffusion kinetics of mEos3.2-ZF5. Single molecule data acquired at an exposure time of 10 milliseconds for meos3.2-ZF5. (Related to Fig. 3).

Movie 5. Volumetric Imaging of ZLD and ZF5 in nc14. 4D volumetric imaging data showing side-by-side comparison of mNeonGreen-ZLD and mNeonGreen-ZF5 during interphase of nc14 pre-cellularization. (Related to Fig. 4).

Movie 6. Volumetric Imaging of ZLD. 4D volumetric imaging data showing mNeonGreen-ZLD through nc12-14. (Related to Fig. 4).

Movie 7. Volumetric Imaging of ZF5. 4D volumetric imaging data showing mNeonGreen-ZF5 through nc12-14. (Related to Fig. 4).

Movie 8. Volumetric Imaging of sfGFP-ZLD. 4D volumetric imaging data showing sfGFP-ZF5 in nc14. (Related to Fig. 4).

Movie 9. Hub Segmentation. Example of 4D hub segmentation for mNeonGreen-ZLD. Zelda-mNeonGreen is shown in gray, and hub masks are shown in fire LUT (purple/red) (Related to Fig. 1 and 4).

Movie 10. Volumetric Imaging of ZF5/+. 4D volumetric imaging data showing mNeonGreen-ZF5/+ through nc12-14. (Related to Fig. 4).

Movie 11. Volumetric Imaging of ΔIDR/+. 4D volumetric imaging data showing mNeonGreen-ΔIDR/+ through nc12-14. (Related to Fig. 4).

Movie 12. Volumetric Imaging of ZF1-6/+. 4D volumetric imaging data showing mNeonGreen-ZF1-6/+ through nc12-14. (Related to Fig. 4).

Movie 13. Simultaneous volumetric Imaging of ZLD in the context of nascent transcription of *Antp*. Volumetric imaging data of mNeonGreen-ZLD (gray) and MCP-mCherry (magenta) localized to

Antp-MS2 in late nc14. Cytoplasmic background of MCP-mCherry is due to the protein lacking a NLS to maximize signal to noise within nuclei. (Related to Fig. 5).

Movie 14. Simultaneous volumetric Imaging of ZF5 in the context of nascent transcription of *Antp* in nc12. Volumetric imaging data of mNeonGreen-ZF5 (gray) and MCP-mCherry (magenta) localized to *Antp-MS2* in nc12. (Related to Fig. 5).

Movie 15. Simultaneous volumetric Imaging of ZF5 in the context of nascent transcription of *Antp* in nc13. Volumetric imaging data of mNeonGreen-ZF5 (gray) and MCP-mCherry (magenta) localized to *Antp-MS2* in nc13. (Related to Fig. 5).

Movie 16. Simultaneous volumetric Imaging of ZF5 in the context of nascent transcription of *Antp* in nc14. Volumetric imaging data of mNeonGreen-ZF5 (gray) and MCP-mCherry (magenta) localized to *Antp-MS2* in nc14. (Related to Fig. 5).
