# Supplementary material for: A fine kinetic balance of interactions directs transcription factor hubs to genes
Source: bioRxiv. 2024 Apr 16:2024.04.16.589811. Preprint. [Version 1] doi: 10.1101/2024.04.16.589811 (PMC11042322; doi:10.1101/2024.04.16.589811)
Supplement: Supplementary file 1 [file media-1.pdf]

Supplementary Information for:

A fine kinetic balance of interactions directs transcription factor hubs to genes

Apratim Mukherjee[1,2,*], Samantha Fallacaro [1,2,3,*], Puttachai Ratchasanmuang[2,4,*], Joseph Zinski[1,2,*], Alan Boka[5], Kareena Shankta[2,6], Mustafa Mir[1,2,4,7†]

1. Department of Cell and Developmental Biology, Perelman School of Medicine, University of Pennsylvania, Philadelphia, PA 19104
2. Center for Computational and Genomic Medicine, Children's Hospital of Philadelphia, Philadelphia, PA 19104
3. Developmental, Stem Cell, and Regenerative Biology Graduate Group, Perelman School of Medicine, University of Pennsylvania, Philadelphia, PA 19104
4. Howard Hughes Medical Institute, Children's Hospital of Philadelphia, Philadelphia, PA 19104
5. Biochemistry and Molecular Biophysics Graduate Group, Perelman School of Medicine, University of Pennsylvania, Philadelphia, PA 19104, USA
6. Roy and Diana Vagelos Program in Life Sciences and Management, University of Pennsylvania, Philadelphia, PA 19104, USA
7. Epigenetics Institute, University of Pennsylvania Perelman School of Medicine, Philadelphia, PA 19104

*Authors contributed equally

†Correspondence to: Mustafa Mir: mirm@chop.edu

Contents of this PDF:

Materials and Methods
Figs. S1 to S11
Tables S1 to S2
Movie Captions
References

Other Supplementary Materials include:

Table S3
Movies S1-S14
Tables S1 to S2

Materials and Methods

Key Resources

Reagent or Resource	Source	Identifier	Additional Information
**Fly Lines**			
mNG-ZLD	Mir et al. 2018 (*1*)	-	Fly line with an N-terminal mNeonGreen fusion tag inserted at the endogenous Zld locus.
mEos3.2-ZLD	Mir et al. 2018 (*1*)	-	Fly line with an N-terminal mEos3.2 fusion tag inserted at the endogenous Zld locus.
sfGFP-ZLD	Hamm et al. (2017) (*2*)		Fly line with an N-terminal sfGFP fusion tag inserted at the endogenous Zld locus.
mNG-ZF5 GLC	This paper	-	Fly line with an N-terminal mNeonGreen fusion tag inserted at the endogenous Zld locus modified with the ZF5 mutation and recombined with a line containing the FRT19A line to generate germline clones for homozygous expression.
mEos3.2-ZF5 GLC	This paper	-	As above but with a mEos3.2 N-terminal tag.
mNG-ΔIDR GLC	This paper	-	As above but with a deletion of all domains N-terminal of the DNA binding domain and with mNeonGreen N-terminal tag
His2B-mEos3.2	Mir et al. 2018 (*1*)	-	Fly line with an H2B-mEos3.2 transgene inserted on chromosome 3. Transgene is expressed ubiquitously under the control of a synthetic tubulin promoter.
His2B-EGFP	Mir et al. 2018 (8)		Fly line with an H2B-eGFP transgene inserted on chromosome 3. Transgene is expressed ubiquitously under the control of a synthetic tubulin promoter.
Antp-ms2	Batut et al. 2022 (*3*)		

**Antibodies**			
Zelda	Eisen Lab (UC Berkeley)	-	
GAF	Lis Lab (Cornell University)		
Anti-GFP antibody	Abcam	ab290	
Anti-beta Tubulin antibody [EPR16774]	Abcam	ab179513	
H3K4me3 Antibody, SNAP-Certified™ for CUT&RUN	EpiCypher	13-0041	
CUTANA™ Rabbit IgG CUT&RUN Negative Control Antibody	EpiCypher	13-0042	
Critical Commercial Reagents			
Concanavalin A Magnetic Beads and Activation Buffer	CellSignaling Technology	93569S	
CUT&RUN pAG-MNase and Spike-In DNAq	CellSignaling Technology	40366S	
NEBNext® Ultra™ II DNA Library Prep Kit for Illumina®	New England Biolabs Inc	E7645 L	
Agencourt AMPure XP	Beckman Coulter	A63880	
Protease Inhibitor Cocktail (PIC)	SigmaAldrich	11697498001	
NEBNext® Ultra™ II RNA Library Prep Kit for Illumina®	New England Biolabs Inc	E7775 S	
D1000 tapestation assay	Agilent	5067-5582	
GlycoBlue™ Coprecipitant	ThermoFisher Scientific	AM9516	
RNA High Sensitivity tapestation assay	Agilent	5067-5579	
Phasemaker™ tubes	ThermoFisher Scientific	A33248	
Nextera XT DNA Library Preparation Kit	illumina	FC-131-1024	
Illumina Tagment DNA Enzyme and Buffer Small Kit	illumina	20034197	
Nextera DNA CD Indexes 24 indexes - 24 samples	illumina	20015881	

TRIzol™ Reagent	ThermoFisher Scientific	15596026	
ECL™ Anti-rabbit IgG, Horseradish Peroxidase	Amersham™	NA934V	
ECL substrate	BioRad	170-5060	
Nitrocellulose membrane	BioRad	1620215	
Digitonin	Milipore	300410	
Spermidine	ThermoFisher	132740010	
**Deposited Data**			
ATAC-seq (WT and ZF5)	This paper	GSE264096	
RNA-seq (WT and ZF5)	This paper	GSE264096	
CUT&RUN (ZLD, ZF5, and GAF)	This paper	GSE264096	
GAF- ChIP-seq	Gaskill et al. (2021) (*4*)	GSE152773	
Dorsal-ChIP-seq	Sun et al. (2015) (*5*)	GSE65441	
RNA microarray	Liang et al. (2008) (*6*)	GSE11231	
**Software**			
Quot	https://github.com/alecheckert/quot	N/A	
SASPT	(*7*) https://github.com/alecheckert/saspt	N/A	
Custom SMT Analysis	https://gitlab.com/mir-lab/publications/ZeldaAnalysis_2024 and 10.5281/zenodo.10905740	N/A	Find all code for this in the "single_molecule_analysis" folder. Gitlab has code only. Zenodo includes data CSVs.
pyEnRICH	https://gitlab.com/mir-lab/publications/pyEnRICH	N/A	
Custom Hub Analysis, MS2-Hub Interaction Analysis	https://gitlab.com/mir-lab/publications/ZeldaAnalysis_2024 and 10.5281/zenodo.10905740	N/A	Find all code for this in the "volumetric_image_analysis" folder. Gitlab has code only. Zenodo includes data CSVs and sample images.
Imaris Parallel Writer	(*64*) https://github.com/abcucberkeley/LLSM5DTools	N/A	
micro-SAM	(*66*) https://github.com/computational-cell-analytics/micro-	N/A	

	sam		
Cellpose 2.0	(*65*) https://github.com/MouseLand/cellpose	N/A	

Fly Husbandry

All germ-line cloned embryos were made in similar fashion using the following protocol. mNG-ZF5/+ flies were expanded in large dextrose food base bottles and kept at 25°C. mNG-ZF5/+ virgins were crossed to males ovoD, hs-FLP FRT-19A at 25°C for 48 hour before the adults were flipped and larvae were heat-shocked at 37°C for 3 consecutive days at 1 hour intervals (Fig. S1A) (*8*). Enclosed mNG-ZF5 adult flies were screened before caged with apple agar plate and fed with yeast paste. Embryos laid overnight from these cages were checked for lethal phenotype for 3 consecutive days before the cages were used. Non heat-shock control bottles were also made to determine ovoD effectiveness or potential non-virgin G0 females. ZF5 mutant embryos fail to gastrulate and begin to show defects in late nuclear cycle 14, consistent with previous observations using complete Zelda knock-out embryos (*6*, *9*).

Western blots

Embryos were collected after incubation for 75 minutes at 25°C. Dechorionated embryos were staged under halocarbon oil 27 and rinsed prior to protein isolation. Around 90-100 embryos were appropriately staged and collected in 1x PBS + PIC. Proteins were extracted under denaturing conditions using a 2x Laemmli SDS sample buffer with 5% BME and heated at 95°C for 5 minutes then chilled as previously described (*10*). Samples were centrifuged at 4°C for 10 minutes at 16000 RPM. Supernatant was placed in new 1.5 ml tubes and stored at -20°C until needed. Protein samples were split into two equal volumes and run on 8% acrylamide gel at 200 volts for 5 hours for zelda protein separation and 1 hr for beta-tubulin separation. Proteins were transferred onto nitrocellulose membranes overnight at 4°C at 40 volts and blocked for 45 minutes with 3% milk in 1X TBST (1X TBS, 0.2% Tween) at room temperature. The blots were then incubated overnight at 4°C with either rabbit anti-Zelda or rabbit anti-beta-tubulin (1:1000 dilution) for loading control. Anti-Rabbit HRP antibody was used as a secondary at 1:5000 for 1 hour at room temperature. Blots were treated with ECL substrate and visualized with the BioRad ChemiDoc system (Fig. S1B).

Embryo collection and mounting for live-imaging

Embryos were collected after incubation at 25°C for 75 minutes. Embryos were dislodged from an apple juice-agar collection plate in water, transferred to a cell strainer, and subjected to bleach for 30-45 seconds to remove the chorion. The plate was then rinsed with distilled water until the bleach odor dissipates, embryos were selected with a fine paintbrush and positioned on an agar pad under a dissection microscope. A 25 mm glass coverslip is made adhesive by depositing 20 µl of a solution of double-sided scotch tape dissolved in heptane. Once the heptane has fully evaporated the embryos are transferred to the coverslip by gently tapping.

Light-sheet microscope optical paths and configuration

The lattice light-sheet microscope (*11*) used in this work is a modified home-built implementation of the instrument similar to the adaptive optics equipped lattice-light sheet (*12*) system built based on designs from the Betzig lab at the HHMI Janelia Research Campus. For experiments in this work the following laser lines were used: 405 nm, 488 nm, and 561 nm. The laser lines were expanded to a diameter of 2 mm combined, passed through a Half-wave plate to adjust polarization and relayed into an acousto-optic tunable filter (Quanta-Tech, AA Opto Electronic) to select wavelength and modulate power. The output from the AOTF was then either sent to an optical path to generate a lattice light-sheet excitation pattern or a multi-gaussian beam excitation pattern. For lattice light-sheet generation, the collimated laser beams were expanded along a single dimension using a Powell Lens (Laserline Optics Canada,) and then width of the expanded beam was adjusted and collimated using a pair of cylindrical lenses. This stripe of collimated light was relayed onto a grayscale Spatial Light Modulator (Meadowlark Optics, AVR Optics) after passing through a second Half-wave plate. The diffracted light from the SLM was then relayed onto a custom annular mask to select the minimum and maximum numerical aperture of the light-sheet and block the undiffracted light from the SLM. The light from the annulus was

demagnified and projected onto resonant galvanometer (Cambridge Technology, Novanta Photonics) conjugate to the sample plane. The resonant galvanometer was used to mitigate shadowing artifacts and other inhomogeneities in the light-sheet by introducing a slight wobble in the excitation angle. The light is then projected onto a pair of galvanometer scanning mirrors (Cambridge Technology, Novanta Photonics) conjugate to the pupil plane for scanning the light-sheet along x and z optical axes in the excitation coordinate plane. Finally, an excitation objective (Thorlabs, TL20X-MPL) was used to focus the light-sheet onto the sample. The emitted fluorescence was collected by a detection objective oriented orthogonally to the excitation objective (Zeiss, 20×, 1.0 NA), and projected onto a Deformable mirror (ALPAO) positioned in a pupil of the detection path. The light was then split using a dichroic beam splitter (Semrock Di03-R561-t3-25x36) and imaged onto two sCMOS detectors (Hamamatsu ORCA Fusion). The first camera had a green emission filter (Semrock FF03-525/50-25) and a notch filter (Chroma ZET488NF) to reject laser light and the second camera had a red emission filter (Semrock FF01-593/46-25) and a notch filter (Chroma ZET561NF) to reject laser light. The detection path optical aberrations were corrected as previously described by adjusting the deformable mirror (Alpao) (*12*).

Volumetric imaging using lattice light-sheet microscopy

Volumetric imaging used for live imaging of Zelda or MCP used a multi-bessel lattice sheet with maximum numerical aperture to minimum numerical aperture ratio of 0.4/0.3 488 nm (used to excite mNeonGreen and sfGFP) and 561 nm (used to excite mCherry) were used for volumetric imaging with laser powers of 0.226 µW and 1.44 µW respectively. Two color channels were acquired simultaneously for a volume of 18.9 µm sampling z-planes spaced 0.3 µm apart with an exposure time of 30 msec. Each volume was acquired every 3 seconds during acquisition.

Single-molecule imaging using light-sheet microscopy

For single-molecule imaging the Gaussian light sheet path was used instead of the lattice light sheet path (see the section on “Light-sheet microscope optical paths and configuration” for more details). Briefly, after passing through the AOTF, the beam was expanded using a Powel lens following which it was relayed onto a custom mask for filtering. The filtered sheet was then projected onto a resonant galvanometer (Cambridge Technology, Novanta Photonics). Finally, an excitation objective (Thorlabs, TL20X-MPL) is used to focus the light-sheet onto the sample. The detection path for the emitted light is the same as described above for the lattice light sheet configuration. It should be noted that for this light path, we bypass the SLM completely in order to ensure higher laser power at the excitation objective.

For all the single-molecule imaging, the 405 nm laser line was kept on constantly during the acquisition period for photoswitching and the 561 nm laser line was used for excitation. Data was acquired at 10 msec and 500 msec exposure times for the fast and slow single-molecule imaging experiments respectively. The excitation laser power was optimized empirically for each exposure time to achieve sufficient contrast for single-molecule tracking and the powers of the photoswitching laser were also optimized empirically to achieve low enough densities of detections to enable tracking. The excitation laser power was 2 mW, 13 mW and switching laser power was 0.2 µW, and 1 µW for 500, and 10 msec exposures, respectively, as measured at the back focal plane of the excitation objective. The same settings were used to acquire control data at each exposure time on His2B-mEos3.2. The number of frames acquired was 8000 and 400 for the 10 msec and 500 msec exposure times respectively corresponding to 80 sec and 200 sec of total imaging time respectively. The acquisition length was optimized such that multiple fields of views could be imaged within each short interphase time while also capturing a sufficient number of trajectories at each position. To optimally position the embryo in the light sheet and to keep track of cell-cycle phase, and nuclear cycle, His2B-eGFP was used.

Processing of single-molecule tracking data

To process the SMT data we first removed the initial frames during which the fluorophores were bleached. Then we took maximum projections of the data to obtain our nuclear mask. Localization and tracking of the single molecule trajectories was performed using the open source software package *quot* (*13*). For the 10 msec data the following key parameters on *quot* were used: (i) for detection - method = “identity”, Spot detection settings = “llr”, k = 1.3, w = 15, t = 20, (ii) for localization - method = “ls_int_gaussian”, window size = 9, sigma = 1.0, ridge = 0.0001, maximum iterations = 10, damp = 0.3, camera gain = 1, camera background = 108 (iii) for tracking - method = “euclidean”, pixel size (in µm) = 0.108, search radius = 0.7 µm, maximum blinking = 0 frames. For the 500 msec data, the following parameters in Quot were used: (i) for detection - method = “identity”, Spot detection

settings = “llr”, k = 1.3, w = 15, t = 23, (ii) for localization - method = “ls_int_gaussian”, window size = 9, sigma = 1.0, ridge = 0.0001, maximum iterations = 10, damp = 0.3, camera gain = 1, camera background = 108 (iii) for tracking - method = “euclidean”, pixel size (in μm) = 0.108, search radius = 0.3 μm, maximum blinking = 0 frames. Once we obtained our list of tracked molecules from quot we filtered them using the nuclear mask as described earlier to ensure we analyzed only trajectories that were occurring inside the nucleus.

Analysis of volumetric imaging data to quantify hub properties

All volumetric imaging was pre-processed prior to downstream analysis pipelines by first conducting GPU-accelerated 3D image deconvolution using CUDA (*14*). All datasets were deconvolved using input PSF taken by bead images on the LLSM and using 5 iterations for Richardson-Lucy based deconvolution. We used a custom Imaris converter leveraging fast [Tiff or Zarr] file readers to generate Imaris files for data visualization and rendering (*15*). After deconvolution, images were subjected to nuclear segmentation. To segment nuclei in our dataset, we created a custom model using Cellpose 2.0 (*16*). Ground truth data was generated using a mixture of micro-Sam (*17*), a napari plugin for segment anything (*18*) and manual correction on wildtype Zelda images. These data were then used to train Cellpose 2.0. The resulting model was then used to segment all slices of each acquired dataset individually. A custom post-processing pipeline was then utilized in order to stitch the individual slices back together and to interpolate any slices of called objects that were missed in segmentation. We then implemented a nearest neighbor algorithm to track nuclei over the course of interphase in each nuclear cycle.

To quantify hub properties, we created a custom analysis pipeline. Nuclei were first normalized to their mean intensity to assess local enrichments of TFs above nuclear background. This pipeline segments hubs by using a median filter to remove noise followed by image erosion and reconstruction. The reconstructed image was subtracted from the median filtered image to first create a binary mask of high density regions. Then, we called local maxima peaks to be used as markers for watershed segmentation in order to separate hubs that might be fused together in the binary mask since we often see hubs as less discrete and more amorphous. We then used region props to quantify different properties of the hubs such as integrated intensity, mean intensity, and size. We then filtered these hubs by their mean enrichment. Using a cumulative distribution function we saw that wild type Zelda hubs reach 0.5 at an enrichment score about 1.4 times the nuclear mean intensity. We used this cut off as a means to clear any hub that might have a minimal and non significant enrichment compared to the nuclear mean intensity (Fig. S6).

To quantify the lifetime of hubs, we used the data acquired after the first 20 frames (~2 min) and before the last 20 frames of each nuclear cycle to avoid confounding effects of chromatin reorganization during early interphase and early prophase. This timeframe is then segmented into a series of 20 frame intervals. At the beginning of each interval, each hub in each nuclei is localized and a 1 μm^3 box is centered around the hub. The mean signal within this box is used to calculate autocorrelations. These autocorrelations were then smoothed using a savgol filter with a window length of 7 and poly order of 2. The zero-crossings were interpolated from the resulting curves and used to measure the hub lifetimes. We verified that lifetimes and hub calling were independent from differences in expression and signal to noise in various mutant backgrounds and embryo to embryo by plotting the hub enrichment and resulting zero-crossing and observed no clear correlation between the two measurements (Fig. S6).

Analysis of volumetric imaging data to quantify hub enrichment at MS2 sites

Preprocessing was completed as stated in the above section for hub analysis. To then quantify the average enrichment of transcription factors and nascent transcription, we created the pyEnRICH package to calculate radial enrichment around the site of transcription to estimate increased local concentration compared to nuclear background as done previously (*1*). This script uses a difference-of-gaussians filter followed by a percentile threshold in order to identify spots of nascent transcription. Then we find non-overlapping random spots in the nucleus to compare to the transcription site. By taking 1.1 μm x 1.1 μm windows we are able to average across time and populations of nuclei in order to estimate the average enrichment using a radial profile of the transcription factor channel centered at the spot of nascent transcription. We remove MS2 sites at the edges of the nucleus to avoid improper results stemming from the boundary of a bright nucleus to low background levels in the cytoplasm. We also included a random nuclear spot control to compare the MS2 site to to assess if hub interactions are random or specific to the locus. To identify a random spot, we orient a 1.5 μm radius from

the center of the MS2 site in a random direction and then ensure this new spot and a radius of 0.6 μm is located fully within the nucleus. We find three non-overlapping random spots per nucleus and average the resulting enrichment from these spots and compare with the MS2 spot.

After using pyEnRICH, we created an additional custom pipeline that interpolates the center MS2 position for any dropped/non-segmented points for all time points after onset of nascent transcription up until the last time point per tracked nucleus segmented. Using these updated positions, we then quantified hub interactions with the MS2 spot and with random sites. Since Zelda hubs, like most Drosophila transcription factors, form amorphous, transient, and almost web-like entities that are often the opposite of discrete, we found that pairwise distance of hub centroid and MS2 centroid coordinates were insufficient to describe interactions. Instead, hub interactions were called based on overlapping segmented hub masks and a 0.5 μm radius sphere centered at either the MS2 or random spot (Fig. 5C). Hubs must be overlapped in the sphere for at least 2 frames to be considered as interacting with the locus as opposed to due to random fluctuations. The duration (length of consecutive time points containing overlapped pixels) and the frequency (amount of hub interactions called in one nucleus over the total time of transcriptional activity) were then quantified and plotted. Additionally, random spots are identified as described above. We assess the hub-interactions between three random spots per nucleus and plot alongside hub-interactions with the MS2 site in order to differentiate between random and specific effects.

Quantification of residence times

Residence times were calculated from single-molecule data acquired at 500 msec exposure times as described previously (*1*, *19*). Briefly, imaging at 500 msec exposures effectively blurs out molecules that are not still for a significant portion of the exposure allowing us to selectively localize and track the bound and slowly diffusing population of molecules. The longer exposure times also allow us to use lower excitation laser powers, thus limited photobleaching and providing longer trajectories. To estimate the genome average residence time survival probability curves were calculated by accumulating data from each experimental condition at each nuclear cycle. An objective threshold of 2 seconds (*1*) was applied to the minimum trajectory length and probabilities below 10^{-3} were not considered for fitting. These filters are applied to remove the effects of tracking errors and slowly diffusing molecules (*20*). The survival probability curves were then fit to a double exponential model of the form $F * \exp(-k_{ns} * t) + (1 - F) * \exp(-k_s * t)$ using the curve_fit function in Python, where k_s is the slower off-rate and k_{ns} is the faster/nonspecific off-rate. An exponential weighting function was used to ensure proper estimation of the slower off-rate. The inferred off-rate k_s is biased by photobleaching and chromatin motion so bias correction was performed as previously described: $k_{s,true}=k_s-k_{bias}$, where k_{bias} is the slower-off rate estimate from fitting the survival probabilities from His2B data. The genome wide residence-times were then calculated as $1/k_{s,true}$. Errors reported were estimated from the standard-deviations of the fit-parameters using standard propagation of error methods.

Analysis of fast single-molecule tracking data

To analyze the 10 msec single molecule trajectories to infer the diffusion kinetics we used a variational Bayesian method called State Array based Single Particle Tracking (*7*). This method does not a priori assume a specific number of diffusive states. Instead, it processes all the recorded trajectories and selects a model that comprehensively describes the trajectories with the minimum combination of state parameters (diffusion coefficient and localization error) as possible. Consequently, it produces average posterior state occupancies for a state array evaluated on the experimental trajectories. For the model selection, it considers a range of diffusion coefficients from 0.01 $\mu m^2/s$ to 100 $\mu m^2/s$ representing the range of physiologically relevant diffusion coefficients reported in the literature. This package is publicly available at https://saspt.readthedocs.io/en/latest/. For our analysis, we used the following specific parameters in the SASPT program:

Likelihood type: RBME
Pixel size: 0.108 (1 pixel = 0.108 μm)
Frame interval: 0.0125433 ms
Concentration parameters: 1
Max iterations: 200
Split Size: maximum trajectory length
Sample Size: number of trajectories

Using SASPT we were able to generate the diffusion coefficient occupancy plots for each protein category both overall and inside the clusters. Furthermore, in order to assign individual diffusion coefficients to each trajectory for downstream analysis, we first weighted the range of diffusion coefficients by their corresponding occupancies for each trajectory. We calculated the geometric mean of these weighted coefficients which was then assigned to each trajectory. Then we segregated the trajectories into three kinetic bins defined as follows: bound trajectories (DC≤0.08 um^2s^{-1}) based on the inflection point from the His2B diffusion coefficient occupancy plot (Fig. S5B), intermediate trajectories (0.08 um^2s^{-1}<DC<0.5 um^2s^{-1}), and fast trajectories (DC≥0.5 um^2s^{-1}) based on the inflection point of the ZLD diffusion coefficient occupancy plot (Fig. S5B).

To determine the clustering behavior from the single molecule trajectories we first calculated the average position of the trajectories by considering the mean x and y locations. Average trajectory positions were considered because we wanted to quantify the diffusion kinetics inside the clusters and hence needed to assign diffusion coefficients to individual trajectories. We then used density-based spatial clustering of applications with noise (DBSCAN) to determine the location of the clusters and the trajectories that comprised the cluster. For the DBSCAN analysis, we specified that there had to be a minimum of 10 points ("min. samples") to define a cluster and the maximum distance between any two points in the cluster was 0.2 µm ("max. distance"). These values were selected by initially plotting the number of clusters as a function of the max. distance to determine where the plot showed an elbow, i.e. after what threshold value of max. distance the number of clusters did not change significantly, a common analysis technique used in DBSCAN to determine the appropriate parameters. The parameters chosen were further validated by qualitatively comparing the DBSCAN predictions to the raw spatial maps of the single molecule trajectories. These values were kept constant between the two protein categories.

For the anisotropy analysis, we first filtered the data sets to remove all the bound trajectories since they can bias the angle distribution. We then calculated the angle between all consecutive jumps using while excluding all jumps that were less than 0.2 µm based on the jump distribution of His2B (Fig. S5D). We then calculated the fold-anisotropy metric as the probability of observing a backward jump (with an angle between jumps in the range [180 - 30,180 + 30]), divided by the probability of observing a forward jump (with an angle between jumps in the range [0 - 30,0 + 30]).

Single molecule trajectories were taken from a minimum of 3 different embryos for both ZLD and ZF5. For calculating the standard deviation for the bound fraction, fold-anisotropy, and average diffusion coefficient, we used bootstrapping to sample 50% of the data 20 individual times. In order to calculate statistical significance where relevant, we used the Mann-Whitney test since our data was not normally distributed as confirmed by the Kolmogorov-Smirnov test.

CUT&RUN

Embryos collection for each genotype varied slightly due to embryo depositing rate especially for germline clone mutant collections, however typical protocol was completed as followed. Embryos were collected at 25°C for 30 minutes, and incubated for 60 minutes at 25°C. Embryos were dechorionated with 50% bleach for 30-45 seconds with vigorous mixing. Embryos were then rinsed thoroughly and moved to slightly moistened apple agar for staging. A small drop of halocarbon oil 27 was overlaid on the embryos to help staging. Properly staged embryos were collected on a small kimwipe paper before gently rinsed with slow drops of water. The embryos were gently rolled off the kimwipe paper and placed on a double-sided tape and placed on a slide. Several pools of CUT&RUN wash buffer (*21*) were placed around the embryos. Using a sterile 18 gauge metal needle, quickly pop embryos while drawing the content into the pools of the wash buffer. Extracted contents were quickly moved in the wash buffer into a 1.5 µl tube and temporarily stored at room temperature while samples were collected. Nuclei were collected by slow centrifugation at room temperature at 1500 RCF for 10 minutes. Nuclei were bound and fragmented using conA beads for 20 minutes at room temperature. Samples were then incubated in primary antibodies at 4°C on a thermomixer with gentle mixing. pAG-MNase was used for fragmentation as directed in manufacturer's protocols. STOP buffer from (*22*) was used for proper chromatin release. Spike-in DNA was added at 2 pg/ml for normalization. CUT&RUN libraries were made using NEBNext Ultra II NGS library kit and size selected using AMPure XP beads using manufacturer's protocols. Each CUT&RUN library were verified using a Tapestation and sequenced by NovoGene Co. with pair-end sequencing at 17M reads-depth per library.

Single-embryo ATAC-seq

Briefly embryos from each genotype were collected for 30 minutes and hand dechorionated after 75 minute incubation at 25°C. Embryos were then mounted on a double-sided tape on a cover slip and overlaid with halocarbon oil 27 for staging. Proper staged nc12-13 embryos were removed off double-sided tape and rinsed before they were placed in 1.5 mL tube caps. Embryos were macerated with an 18-gauge needle in lysis buffer (10 mM Tris-HCl pH7.5; 10 mM NaCl, 3 mM $MgCl_2$; 0.1% Igepal-630). Samples were quickly spun and placed on dry-ice to snap freeze. Fragmentation and ATAC-seq library amplifications were performed as previously described (*23*, *24*)).

CUT&RUN and ATAC-seq analysis

For ATAC-seq and CUT&RUN, reads were aligned to the Berkeley Drosophila Genome Project (version six) reference genome using bowtie2 with the '--sensitive-local' setting. Samtools was used to convert from .sam to .bam, index, sort, and remove duplicate, unmapped, and multi-mapped reads. For Cut and Run, peaks were called using macs2 using the '-f BAMPE', '-g 129789873', and '--bw 300' settings using IgG as a control. For ATAC-seq, no IgG was used as a control and --nomodel --nolambda settings were used. Consensus peaks were called using the 'diffbind' package in R. Peaks were annotated using the CHIPseeker package and 'TxDb.Dmelanogaster.UCSC.dm6.ensGene' annotation. Bigwig files were generated using the Bamcoverage function from the *deeptools* package using the a '--binSize 20', '--smoothLength 60', '--centerReads', '--normalizeUsing RPKM', '--effectiveGenomeSize 142573017'. Bigwigs were further normalized by subtracting the chromosomal median from all bins. WT and ZF5 conditions were averaged using bigwigAverage with a '-bs 20'. Heatmaps were generated using deepTools computeMatrix and plotHeatmap functions (*25*). Profile plots were generated using deeptools plotProfile function

Motif analysis

Motif counts within peaks were generated for Zelda, Dorsal, and Gaf. The motif matrices were obtained from JASPAR with the identifiers MA1462.1 (Zld), MA0205.2 (Trl/Gaf), and MA0023.1 (Dl). Peaks called from the Zelda CUT&RUN were sorted into unique to WT, unique to Zf5, and consensus. Peaks were expanded by 50 basepairs at each end. Motifs falling within these peak regions with a match fraction higher than 0.9 for Dl and Zld and 0.85 for Trl/Gaf were summed for each condition. Histograms of the number of counted motifs per peak were made.

RNA-seq

Embryos were collected after 30 minutes of laying time and incubated for 75 minutes at 25°C. Embryos were hand dechorionated and mounted onto a double-sided tape and overlaid with halocarbon oil 27. Each embryo was staged to proper nc12 to nc13 then rinsed before they were placed on 1.5 mL microcentrifuge tube caps (3 embryos per cap). Embryos were macerated in 10 µl TRIzol™ using sterile an 18 gauge needle. The caps were then closed and stored on ice until all samples were collected. Samples were briefly spun down and additional Trizol were added. Samples were incubated at room temperature for 5 minutes before equal volume of chloroform was added. This mixture was quickly spun down and moved to Phasemaker™ tubes. Isolation of total RNA was completed as previously described (*26*) with a small modification where GlycoBlue was added according to manufacturer's instructions as a coprecipitant to help visualize RNA pellets. Isolated total RNA samples were measured for quality using RNA High Sensitivity tapestation assay; samples with RIN 9 or above were used to make final RNA libraries. RNA-seq libraries were made using NEBNext® Ultra™ II RNA Library Prep Kit for Illumina® following manufacturer's recommended protocol. Libraries' quality were checked using D1000 tapestation assay. Single embryo libraries were sequenced by NovoGene Co. using pair-ended sequencing at 27M reads-depth per library.

RNA-seq analysis

To quantify transcript abundances from RNA-seq data we used the pseudoaligner Kallisto. Reads were aligned to the *Drosophila melanogaster* BDGP6.28 cDNA reference using a pre-built kallisto index (Bray et al., 2016). Data was normalized by eliminating transcripts with no reads in more than half of the samples from each condition and then using EdgeR to perform the 'Trimmed Mean of M-values' method to correct for compositional differences between libraries. Clear differences between the WT and ZF5 transcript abundances were observed via Principal Component Analysis (Fig S3). To determine differentially expressed genes, the 'voom' method from the 'Limma' package in R was applied to the log-transformed counts per million (CPM) data. After fitting linear

models to the transformed gene expression data using the lmFit function, we applied the empirical Bayes (eBayes) method to the model fits. Subsequently, we extracted the top DEGs from the eBayes-fitted models. We adjusted for multiple testing to control the false discovery rate using the Benjamini-Hochberg (BH) method. Genes were classified as differentially expressed if they had logFC greater than 1.5 and an adj. p-value less than 0.05.

To analyze the microarray data from Liang et al. (*6*), we imported the microarray data using the \`ReadAffy\` function from the \`affy\` package to handle .CEL.gz files, which were then normalized employing the Robust Multi-array Average (RMA) method. To identify differentially expressed genes, the \`limma\` package was used to fit linear models to the expression data. The empirical Bayes (eBayes) method was applied to enhance the statistical power and adjust for multiple testing using the Benjamini-Hochberg (BH) method, similar to our RNA-seq data analysis approach. We integrated the CUT&RUN, ATAC-seq, and RNA-seq data sets at promoters and across gene bodies in WT and ZF5 embryos (Table S3). Reads within 2000 bp of TSS of a gene were counted as promoters. Reads across the gene body plus 500 bp at the 5' and 3' end were counted as gene body.

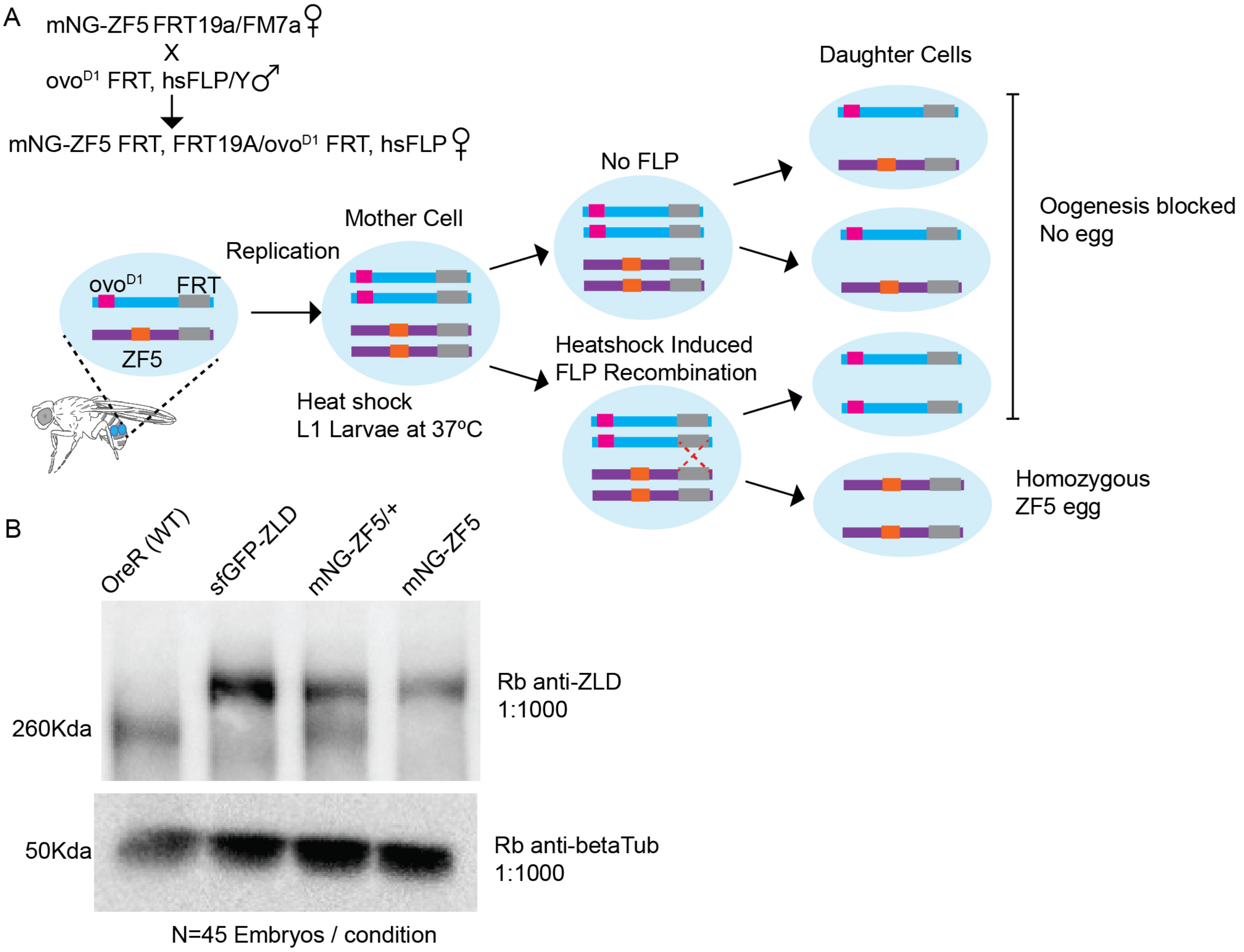

Fig S1: (A) Scheme for generation of germline clones containing homozygous mutations for Zelda using the FLP/FRT system (figure adapted from https://bdsc.indiana.edu/stocks/recombinases/dfs.html). **(B)** Western blots to assess if any wild type Zelda protein is present in embryos generated from germline clones.

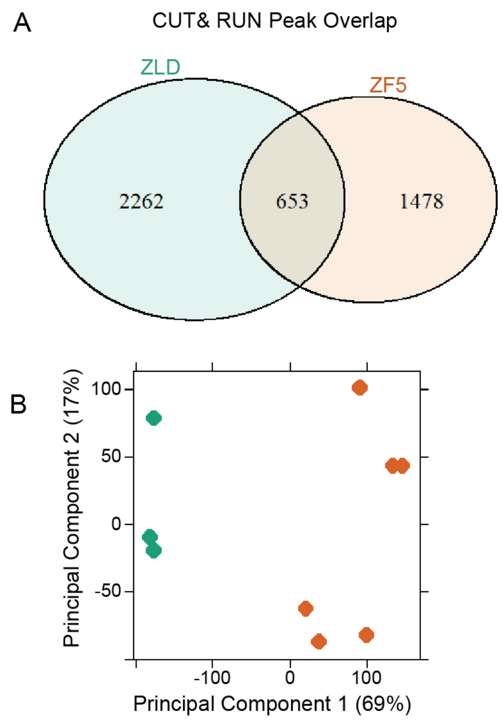

Fig. S2. Differences in CUT&RUN signals (A) A comparison of overlap in identified peaks in CUT&RUN experiments on Wildtype and ZF5 embryos against Zelda. **(B)** Principal component analysis of the replicates from which peaks were calculated in (A).

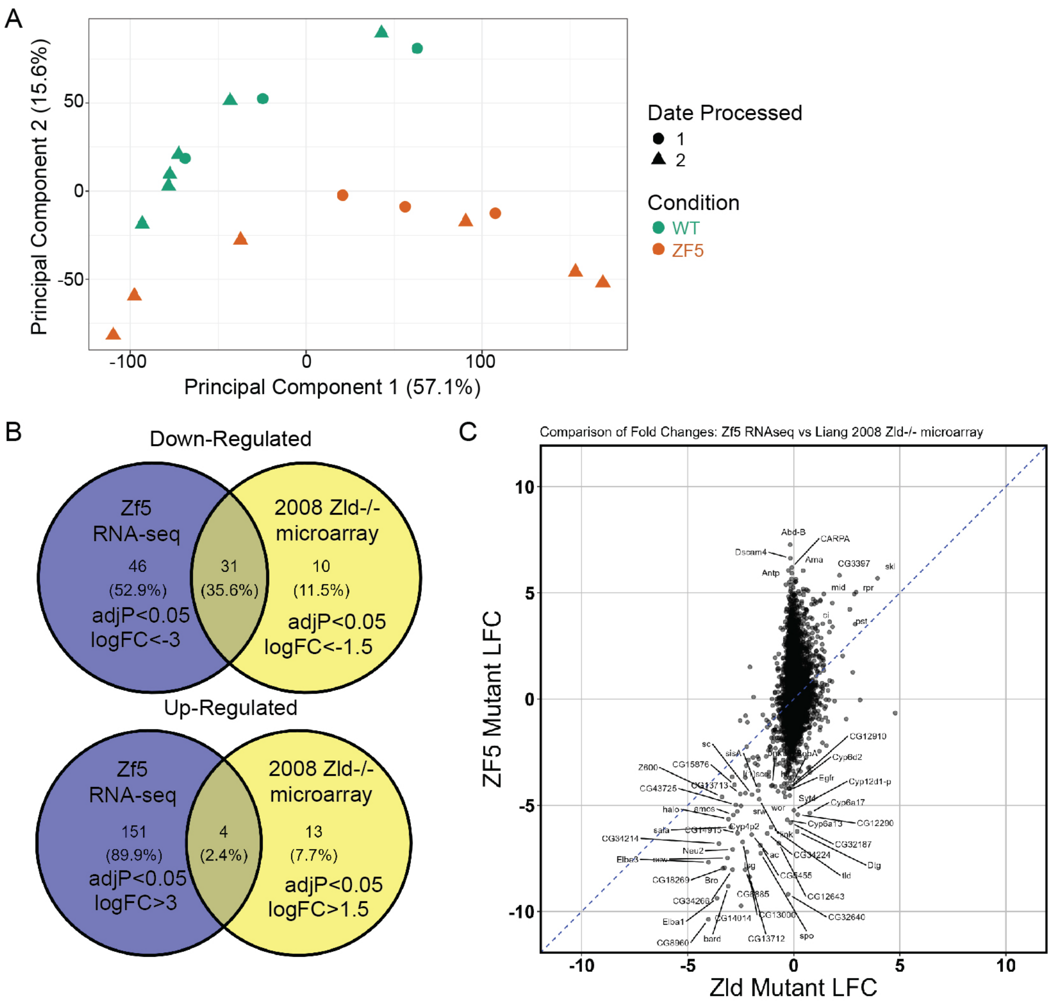

Fig. S3. **Gene expression analysis**. **(A)** Principal component analysis on RNA-seq data. **(B)** Overlap between genes in our RNA-seq data and Zelda null mutant microarray data that meet LFC and adjusted P value cutoffs shown (*6*). **(C)** A scatter plot of all ZF5 RNA-seq gene expression data plotted against the ZLD null mutant microarray data.

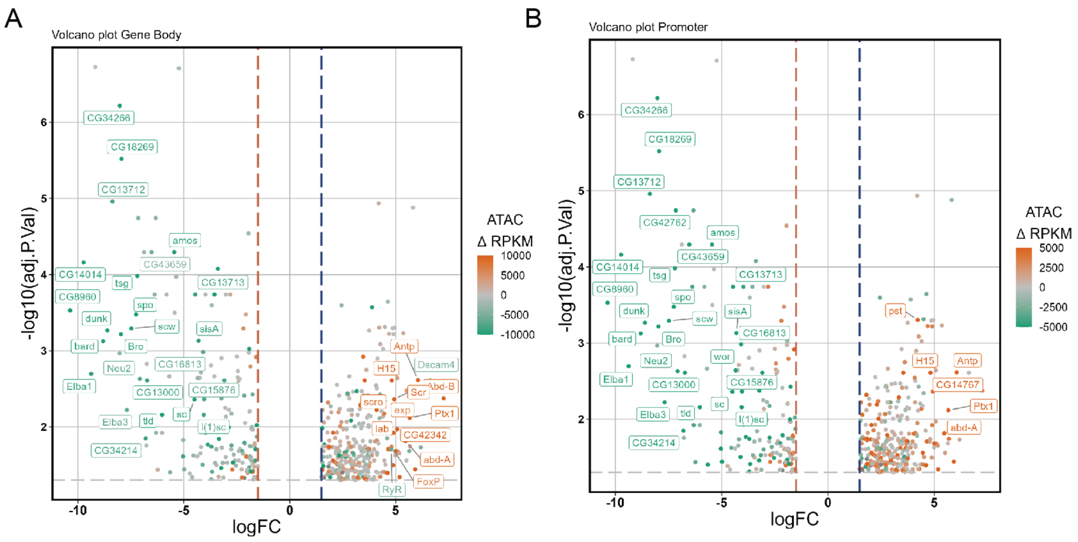

Fig. S4 Changes in accessibility at up- and down-regulated genes (A) Volcano plots of genes up- and down-regulated in ZF5 mutant measured via RNA-seq. Genes with a significance <0.01 and Log Fold Change > 4 and a difference in RPKM > 5000 (From CUT&RUN) are labeled . The gray dotted line marks adjust P-value = 0.05, blue and red dotted lines are +/- 1.5 LCF. Points are colored by the difference in Zelda RPKM between WT and ZF5 mutants across the gene body of each gene. **(B)** Points are colored by the difference in ATAC RPKM between WT and ZF5 mutants within 2kb of the TSS.

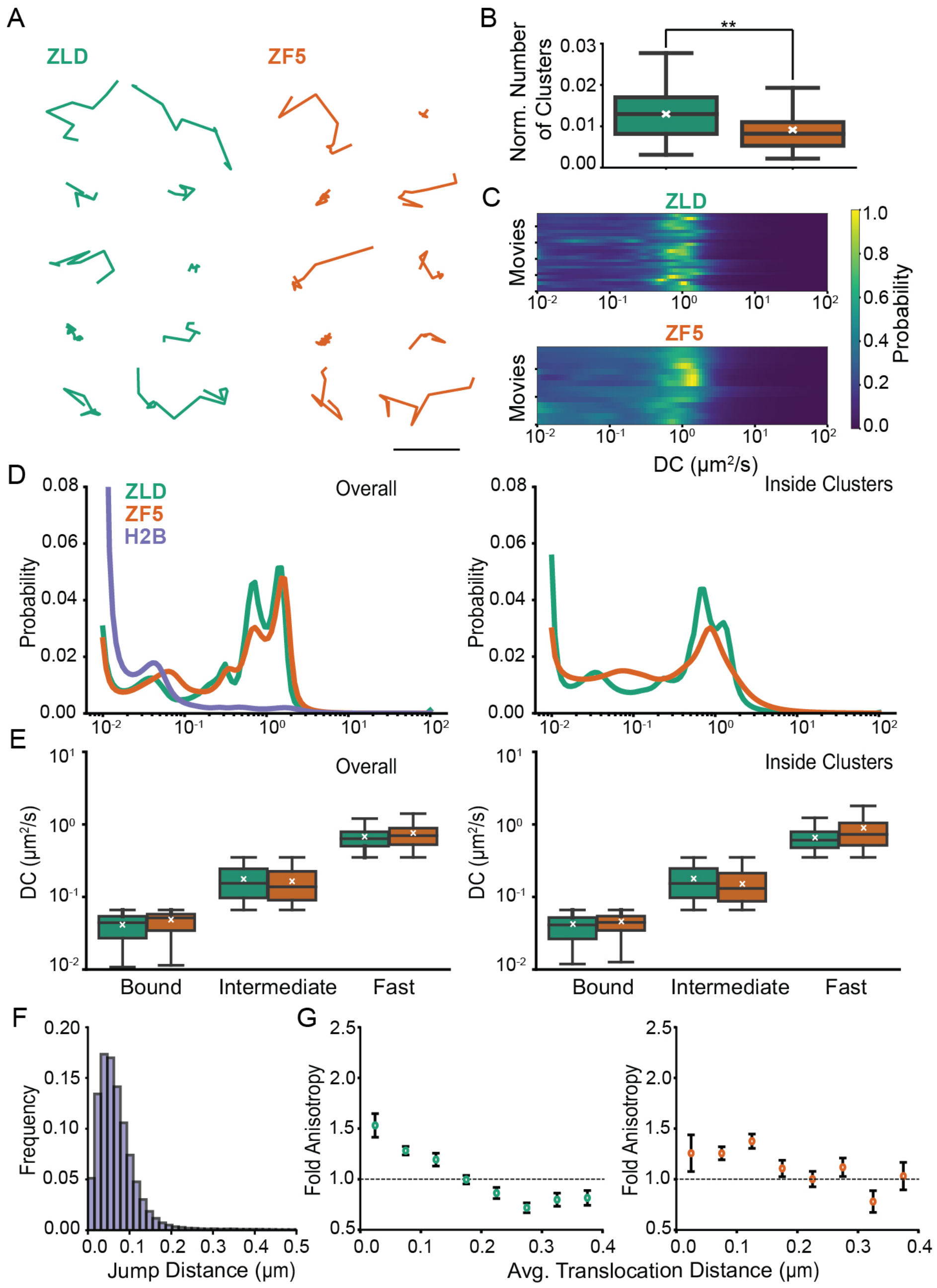

Fig. S5 Fast single molecule tracking (SMT) data analysis (A) Example SMT trajectories at 10 msec exposure times for ZLD and ZF5. Scale bar is 300 nm. **(B)** Quantification of cluster numbers normalized by the total trajectories per nucleus for ZLD (n=172) and ZF5. (n=84). **(C)** Heatmaps of the probability spectra of diffusion coefficients obtained for each movie (one movie/row). Data was analyzed over 23 movies from 7 embryos for ZLD and 14 movies from 3 embryos for ZF5. **(D)** Diffusion coefficient spectra for ZLD, ZF5 and H2B for all trajectories (left) and for ZLD and ZF5 trajectories inside clusters (right). A total of 70,827, 40,307, and 51,158 tracks were measured for ZLD, ZF5 and H2B cases respectively overall, and 14,516 tracks and 3,375 tracks were measured for ZLD and ZF5 respectively in clusters. **(E)** Quantification of the average diffusion coefficient in each kinetic bin for ZLD and ZF5 overall (left) and inside clusters (right). White cross is the mean, error bars represent standard deviations from bootstrapping analysis. **(F)** Histogram of jump distances from H2B trajectories used to determine the distance threshold for anisotropy analysis. **(G)** Fold anisotropy as a function of average translocation distance (over two consecutive jumps) for ZLD (left) and ZF5 (right) overall. Error bars represent standard deviation from bootstrapping analysis. Dashed line indicates an isotropic distribution.

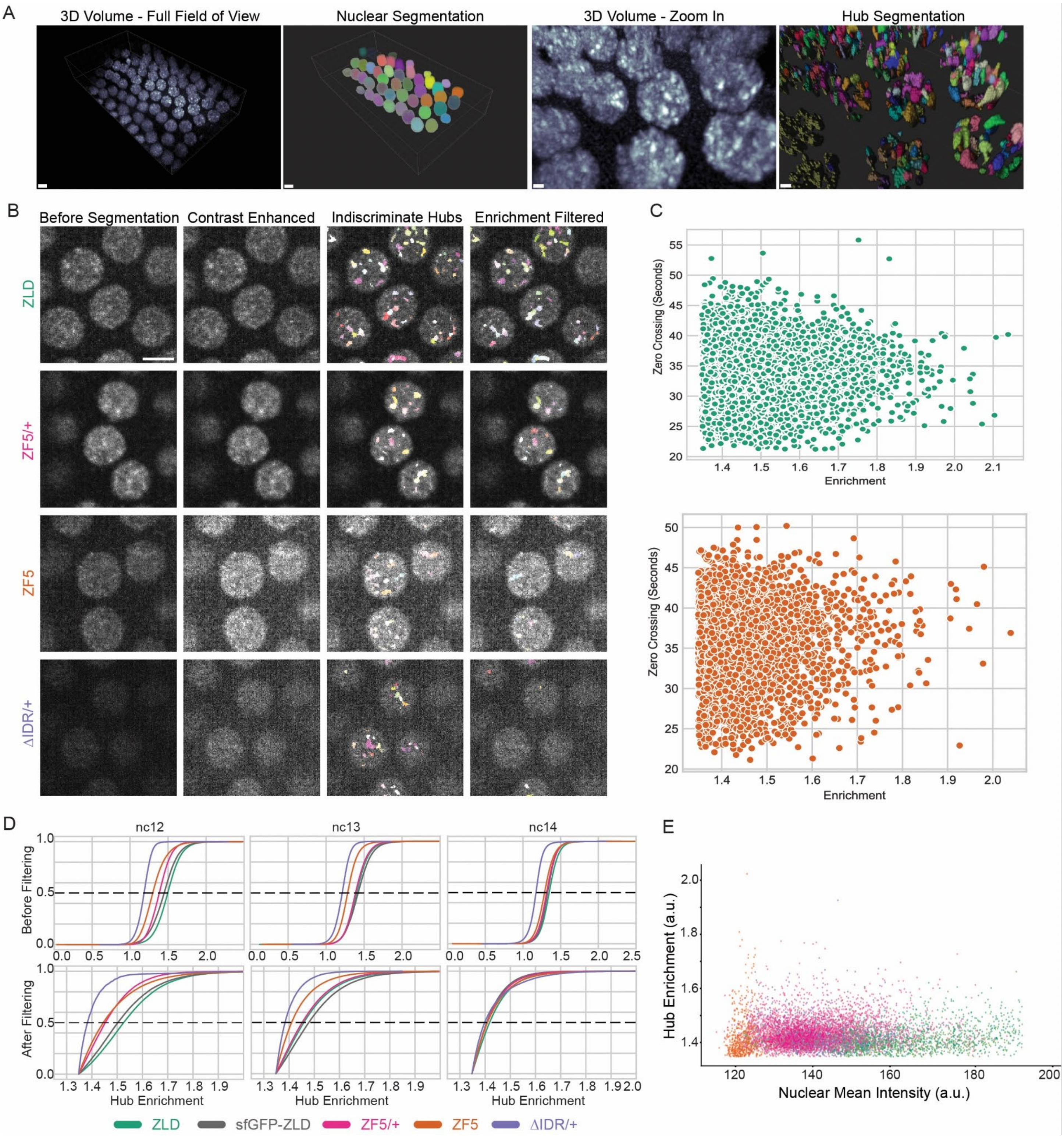

Fig. S6: Analysis of Zelda hubs across mutants. (A) Representation of hub analysis: whole volumes of a single layer of nuclei on the *Drosophila* embryo surface are acquired during imaging and deconvolved (left). Scale bar = 5 µm. Then nuclei are segmented in 3D using a trained machine learning model (middle left). Scale bar = 5 µm. Zelda shows non-uniform distributions within single nuclei (middle right). Scale bar = 1 µm. Hub segmentation is performed in 3D using a custom analysis pipeline (right). Scale bar = 1 µm. **(B)** Visualization of all Zelda mutants (ZLD, ZF5/+. ZF5, ΔIDR/+) before segmentation, with contrast enhancements, after initial segmentation when hubs are indiscriminately called, and after filtering when hubs are kept if they are above the hub enrichment score at 0.5 cumulative probability of the wildtype ZLD.

Each image is a single z-slice and the scale bar is 5 μm. **(C)** Quantification of hub enrichment along with the zero crossing in seconds representing the hub lifetime show no correlation in either ZLD (top) or ZF5 (bottom) between enrichment and lifetime of each hub. **(D)** Cumulative distributions of hub enrichment for each Zelda mutant before and after filtering (top and bottom respectively). **(E)** Scatter plot showing hub enrichment as a function of nuclear mean intensity. Hub enrichment is not impacted by signal-to-noise difference among mutants. N values for nuclei and embryos are consistent with Fig. 1.

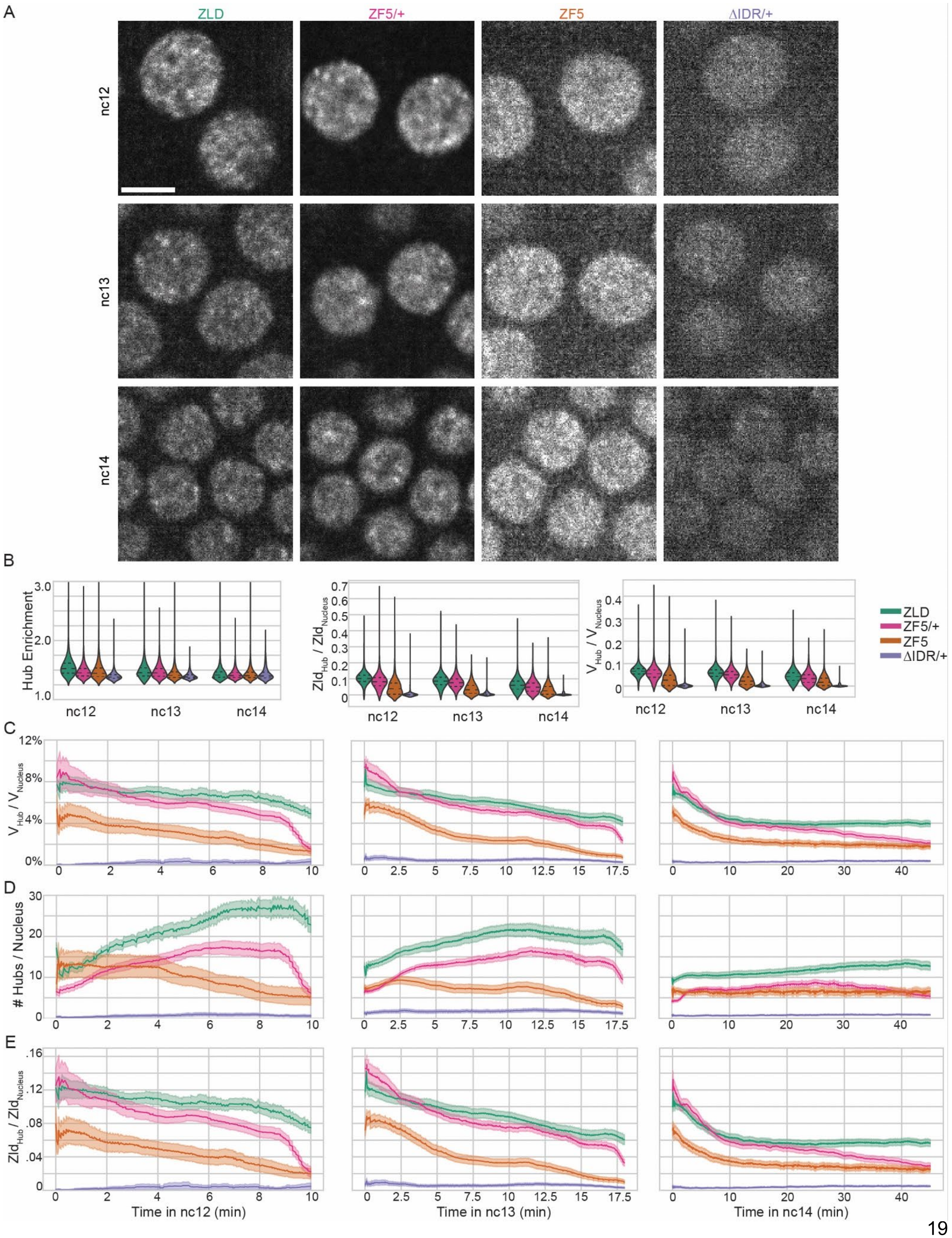
A
ZLD
ZF5/+
ZF5
ΔIDR/+
nc12
nc13
nc14
B
Hub Enrichment
Zld_{Hub} / $Zld_{Nucleus}$
V_{Hub} / $V_{Nucleus}$
ZLD
ZF5/+
ZF5
ΔIDR/+
C
V_{Hub} / $V_{Nucleus}$
D
Hubs / Nucleus
E
Zld_{Hub} / $Zld_{Nucleus}$
Time in nc12 (min)
Time in nc13 (min)
Time in nc14 (min)

Fig. S7: Quantification of biophysical properties of Zelda hubs in different mutants. (A) Representative single z-slices of nuclei in nc12, nc13, and nc14 for all Zelda mutants. Each image is contrast enhanced for visualization. **(B)** Quantification of biophysical hub properties including hub enrichment (mean hub intensity normalized using mean nuclear intensity), number of hubs segmented per nucleus, proportion of Zelda within hubs (sum intensity) compared to all nuclear Zelda (sum intensity), and the volume fraction of hubs within the nucleus through nc 12, 13, and 14. Due to the amorphous and rarely discrete appearance of Zelda hubs, the overall quantity of hubs is more reliably captured in their total volume fraction as opposed to the number of hubs. N values for nuclei and embryos quantified are consistent with Fig. 4. A Kruskal-Wallis test followed by Mann-Whitney U-tests were performed for all pairs within each nuclear cycle. Every pairing is significant ($p<0.01$) except for hub enrichment in nc14 where there is no significant difference in enrichment between any of the mutant pairings. **(C)** Volume fraction of hubs within the nucleus through time in interphase in nc12, 13, and 14. Decrease in volume fraction during the nuclear cycle is attributed to the increase in nuclear volume during interphase. Standard error between embryo replicates is plotted along with the mean. **(D)** Average number of hubs per nucleus and standard error across embryo replicates is plotted throughout the interphase of nc12, 13, and 14. **(E)** Proportion of Zelda within hubs and standard error across embryo replicates is plotted throughout the interphase of nc12, 13, and 14.

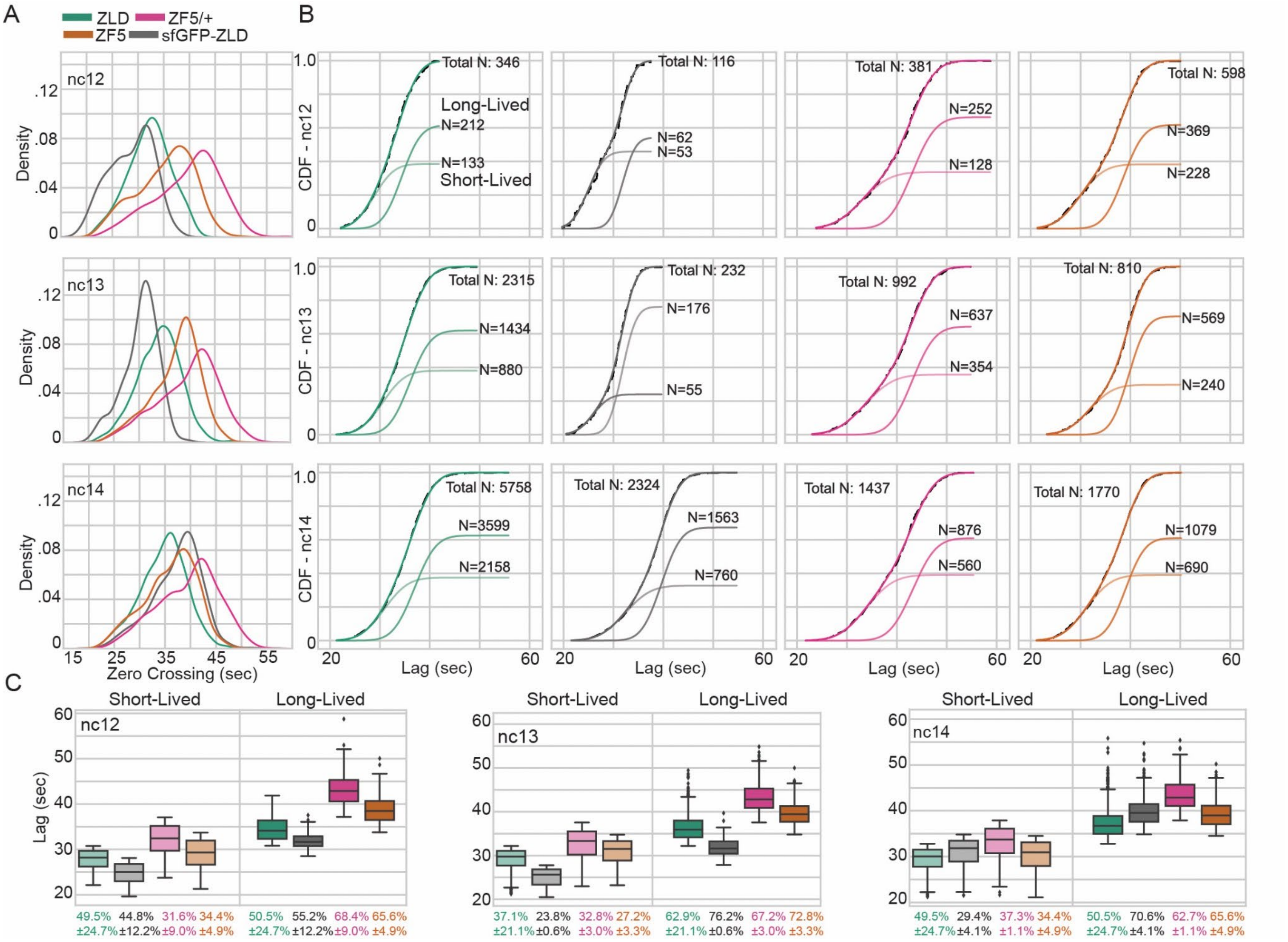

Fig. S8: Hub lifetimes quantified across Zelda mutants. (A) Density plots for Zelda mutants showing zero crossing of the autocorrelation of intensity of a 1 μm^3 box centered at each hub. Distributions are not normal but suggest a two-component mixture of hubs. **(B)** Cumulative distributions of zero crossings from original data (black dotted line), fitted CDF (solid dark color), and two gaussian components (lighter colors). N values provided for the number of hubs fit into these components. For nc12, total N is derived from hubs within 72 ZLD nuclei from 4 embryos, 58 sfGFP-ZLD nuclei from 2 embryos, 341 ZF5 nuclei from 5 embryos, and 59 ZF5/+ nuclei from 3 embryos. For nc13, total N is derived from hubs within 312 ZLD nuclei from 9 embryos, 51 sfGFP-ZLD nuclei from 2 embryos, 127 ZF5 nuclei from 4 embryos, and 236 ZF5/+ nuclei from 3 embryos. For nc12, total N is derived from hubs within 895 ZLD nuclei from 13 embryos, 188 sfGFP-ZLD nuclei from 3 embryos, 445 ZF5 nuclei from 5 embryos, and 236 ZF5/+ nuclei from 3 embryos. **(C)** Zero-crossing distributions shown in box-plots from each component (short- and long-lived) for nc12, nc13, and nc14 for Zelda mutants. Proportion of hubs within each of the conditions is as written underneath each box plot. A Kruskal-Wallis test followed by Mann-Whitney U Test performed among all pairings in the same component in the same nc. All pairing are significant ($p<0.01$) except for between long-lived ZF5 and sfGFP-ZLD hubs in nc14.

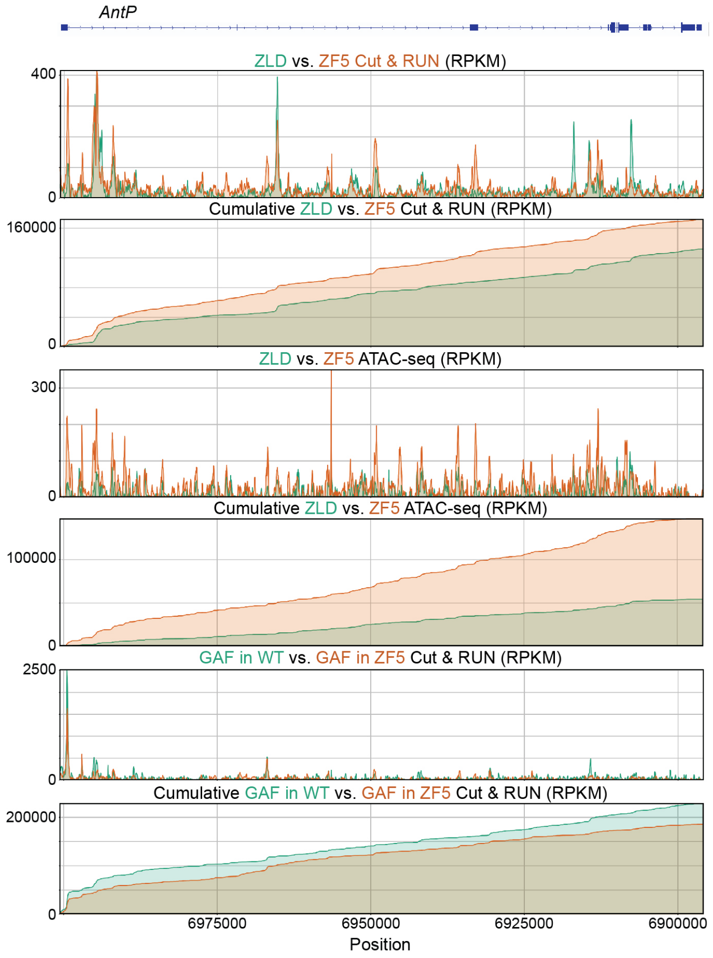

Fig. S9. Measured and Cumulative RPKM from CUT&RUN against ZLD and GAF, and ATAC-seq, in WT and ZF5 embryos, along the *AntP* locus.

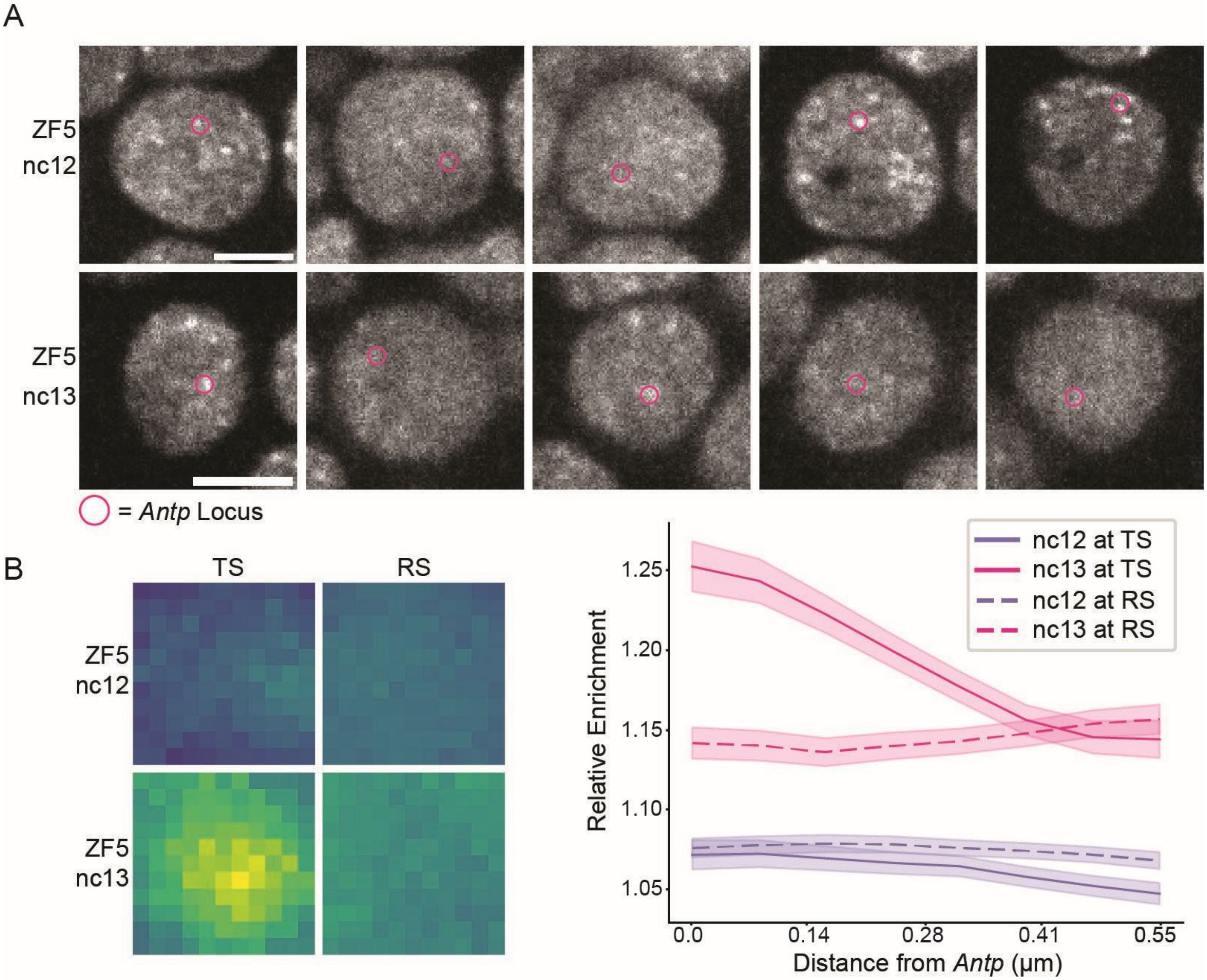

Fig S10: Ectopic expression of *Antp* in nc12 and nc13 in ZF5. (A) Representative images (single slice in Z) of 5 different nuclei for ZLD and ZF5. Zelda is shown in grayscale while the location of the MCP-mCherry at *Antp-MS2* locus is shown as a pink circle on the images. Scale bar is 5μm. **(B)** Cropped images show average intensity centered either at the transcription site (TS) or random site (RS) in the nucleus. Image size is 1.1 μm x 1.1 μm. All cropped images have the same contrast adjustment. Average radial profile with standard error centered at the *Antp* shows Zelda ZF5 in either nc12 (purple) or nc13 (pink) enrichment as a function of distance from MS2 spot center. Two embryos were analyzed in nc12 and one in nc 13.

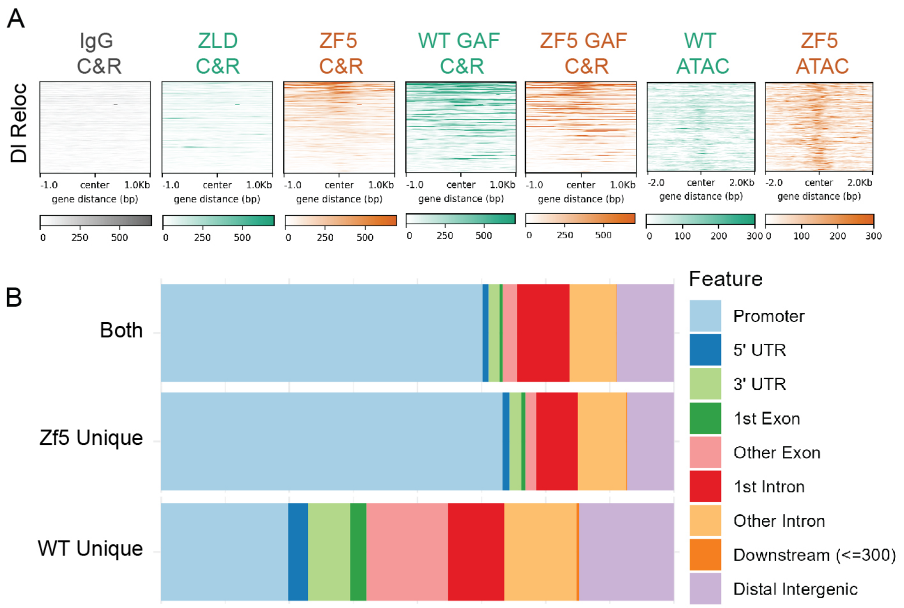

Fig.S11 (A) ZLD and GAF CUT&RUN (C&R), and ATAC-seq in WT and ZF5 at sites of Dorsal relocalization in *zld* null embryos as identified in Sun et al. 2015 (*5*). **(B)** Annotation of peak call locations.

Table S1. Summary of RNA-seq experiments and associated file names submitted to GEO

Sample/Filename	Condition	Day	Genotype	Read Count
H2B_GFPwt1_1_1	WT	2	H2B-eGFP (III)	21044634
H2B_GFPwt2_2_1	WT	2	H2B-eGFP (III)	17390264
H2B_GFPwt2_3_1	WT	2	H2B-eGFP (III)	17693844
H2B_GFPwt2_5_1	WT	2	H2B-eGFP (III)	23905163
H2B_GFPwt3_1	WT	2	H2B-eGFP (III)	16472166
H2B_GFPwt4_1	WT	2	H2B-eGFP (III)	21418487
znf5mtGLC2_1_1	Zf5	2	mNGzld_znf5mt_FRT19A/mNGzld_znf5mt_FRT19A	13801043
znf5mtGLC2_6_1	Zf5	2	mNGzld_znf5mt_FRT19A/mNGzld_znf5mt_FRT19A	20463584
znf5mtGLC2_8_1	Zf5	2	mNGzld_znf5mt_FRT19A/mNGzld_znf5mt_FRT19A	13055948
znf5mtGLC2_9_1	Zf5	2	mNGzld_znf5mt_FRT19A/mNGzld_znf5mt_FRT19A	15760098
znf5mtGLC2_12_1	Zf5	2	mNGzld_znf5mt_FRT19A/mNGzld_znf5mt_FRT19A	29243336
znf5mtGLC2_13_1	Zf5	1	mNGzld_znf5mt_FRT19A/mNGzld_znf5mt_FRT19A	20783123
mNGznf5_GLC1_1	Zf5	1	mNGzld_znf5mt_FRT19A/mNGzld_znf5mt_FRT19A	19518017
mNGznf5_GLC2_1	Zf5	1	mNGzld_znf5mt_FRT19A/mNGzld_znf5mt_FRT19A	18432907
mNGznf5_GLC3_1	Zf5	1	mNGzld_znf5mt_FRT19A/mNGzld_znf5mt_FRT19A	23425471
sfGFPzldwt_1B_1	WT	1	sfGFPzld-wt FD1	21583776
sfGFPzldwt_3_1	WT	1	sfGFPzld-wt FD1	13681957

sfGFPzldwt_5B_1	WT	1	sfGFPzld-wt FD1	23554319

Table S2. Summary of CUT&RUN and ATAC-seq experiments and associated file names submitted to GEO

Filename	Condition	Antibody	Assay	Date	Total Reads	% Aligned w/Bowtie2	Aligned Reads	RiP
ATAC_H2B	WT	NA	ATAC	230719	28344155	87.88	24908844	0.29
ATAC_Znf5_1	ZF5	NA	ATAC	230719	29298587	84.07	24631323	0.35
ATAC_Znf5_2	ZF5	NA	ATAC	230719	30503525	81.33	24808517	0.31
sfGFPzldwt_Gaf1	WT	Gaf	C&R	240323	7955238	16.95	1348413	0.28
sfGFPzldwt_Gaf2	WT	Gaf	C&R	240323	11662950	14.26	1663137	0.29
GLC_mNGzld_Gaf_1	ZF5	Gaf	C&R	231207	15290910	81.98	12535489	0.23
GLC_IgG_1	ZF5	IgG	C&R	230620	14094365	90.45	12748354	0.04
GLC_IgG_2	ZF5	IgG	C&R	231207	17240038	55.5	9568222	0.04
sfGFPzldwt_zld3	WT	Zld	C&R	231207	18168704	86.64	15741366	0.07
sfGFPzldwt_zld4	WT	Zld	C&R	231207	22504068	94.11	21178579	0.14
sfGFPzldwt_zld5	WT	Zld	C&R	231207	22142523	95.7	21190395	0.15
mNGzld_GLC_1	ZF5	Zld	C&R	230620	19287444	86.89	16758861	0.18
mNGzld_GLC_2	ZF5	Zld	C&R	230620	24786487	91.14	22590405	0.08
mNGzld_GLC_3	ZF5	Zld	C&R	230719	18340650	93.03	17062307	0.08
mNGzld_GLC_4	ZF5	Zld	C&R	231207	19221901	73.22	14074276	0.08
mNGzld_GLC_5	ZF5	Zld	C&R	231207	13504968	49.79	6724124	0.19

mNGzld_GLC_6	ZF5	Zld	C&R	231207	12479679	47.82	5967783	0.21

Table S3 Summary statistics of differentially expressed genes (download .csv from supplementary materials)

MOVIE CAPTIONS

Movie 1. Single-Molecule Imaging for determining residence times. Single molecule data acquired at an exposure time of 500 milliseconds for mEos3.2-His2B, mEos3.2-ZLD, and mEos3.2-ZF5. (Related to Fig. 2).

Movie 2. Single-Molecule Imaging for quantifying diffusion kinetics of mEos3.2-ZLD. Single molecule data acquired at an exposure time of 10 milliseconds for mEos3.2-ZLD (Related to Fig. 3).

Movie 3. Single-Molecule Imaging for quantifying diffusion kinetics of mEos3.2-ZF5. Single molecule data acquired at an exposure time of 10 milliseconds for meos3.2-ZF5. (Related to Fig. 3).

Movie 4. Volumetric Imaging of ZLD and ZF5 in nc14. 4D volumetric imaging data showing side-by-side comparison of mNeonGreen-ZLD and mNeonGreen-ZF5 during interphase of nc14 pre-cellularization. (Related to Fig. 4).

Movie 5. Volumetric Imaging of ZLD. 4D volumetric imaging data showing mNeonGreen-ZLD through nc12-14. (Related to Fig. 4).

Movie 6. Volumetric Imaging of ZF5. 4D volumetric imaging data showing mNeonGreen-ZF5 through nc12-14. (Related to Fig. 4).

Movie 7. Volumetric Imaging of sfGFP-ZLD. 4D volumetric imaging data showing sfGFP-ZF5 in nc14. (Related to Fig. 4).

Movie 8. Volumetric Imaging of ZF5/+. 4D volumetric imaging data showing mNeonGreen-ZF5/+ through nc12-14. (Related to Fig. 4).

Movie 9. Volumetric Imaging of ΔIDR/+. 4D volumetric imaging data showing mNeonGreen-ΔIDR/+ through nc12-14. (Related to Fig. 4).

Movie 10. Hub Segmentation. Example of 4D hub segmentation for mNeonGreen-ZLD. Zelda-mNeonGreen is shown in grey, and hub masks are shown in fire LUT (purple/red) (Related to Fig. 4).

Movie 11. Simultaneous volumetric Imaging of ZLD in the context of nascent transcription of *Antp*. Volumetric imaging data of mNeonGreen-ZLD (gray) and MCP-mCherry (magenta) localized to *Antp-MS2* in late nc14. (Related to Fig. 5).

Movie 12. Simultaneous volumetric imaging of ZF5 in the context of nascent transcription of *Antp* in nc12. Volumetric imaging data of mNeonGreen-ZF5 (gray) and MCP-mCherry (magenta) localized to *Antp-MS2* in nc12. (Related to Fig. 5).

Movie 13. Simultaneous volumetric imaging of ZF5 in the context of nascent transcription of *Antp* in nc13. Volumetric imaging data of mNeonGreen-ZF5 (gray) and MCP-mCherry (magenta) localized to *Antp-MS2* in nc13. (Related to Fig. 5).

Movie 14. Simultaneous volumetric imaging of ZF5 in the context of nascent transcription of *Antp* in nc14. Volumetric imaging data of mNeonGreen-ZF5 (gray) and MCP-mCherry (magenta) localized to *Antp-MS2* in nc14. (Related to Fig. 5).

SUPPLEMENTARY REFERENCES

1. M. Mir, M. R. Stadler, S. A. Ortiz, C. E. Hannon, M. M. Harrison, X. Darzacq, M. B. Eisen, Dynamic multifactor hubs interact transiently with sites of active transcription in Drosophila embryos. *Elife* **7** (2018).
2. D. C. Hamm, E. D. Larson, M. Nevil, K. E. Marshall, E. R. Bondra, M. M. Harrison, A conserved maternal-specific repressive domain in Zelda revealed by Cas9-mediated mutagenesis in Drosophila melanogaster. *PLoS Genet.* **13**, e1007120 (2017).
3. P. J. Batut, X. Y. Bing, Z. Sisco, J. Raimundo, M. Levo, M. S. Levine, Genome organization controls transcriptional dynamics during development. *Science* **375**, 566–570 (2022).
4. M. M. Gaskill, T. J. Gibson, E. D. Larson, M. M. Harrison, GAF is essential for zygotic genome activation and chromatin accessibility in the early embryo. *Elife* **10** (2021).
5. Y. Sun, C.-Y. Nien, K. Chen, H.-Y. Liu, J. Johnston, J. Zeitlinger, C. Rushlow, Zelda overcomes the high intrinsic nucleosome barrier at enhancers during Drosophila zygotic genome activation. *Genome Res.* **25**, 1703–1714 (2015).
6. H.-L. Liang, C.-Y. Nien, H.-Y. Liu, M. M. Metzstein, N. Kirov, C. Rushlow, The zinc-finger protein Zelda is a key activator of the early zygotic genome in Drosophila. *Nature* **456**, 400–403 (2008).
7. A. Heckert, L. Dahal, R. Tjian, X. Darzacq, Recovering mixtures of fast-diffusing states from short single-particle trajectories. *Elife* **11** (2022).
8. T. B. Chou, N. Perrimon, The autosomal FLP-DFS technique for generating germline mosaics in Drosophila melanogaster. *Genetics* **144**, 1673–1679 (1996).
9. M. Mir, A. Reimer, J. E. Haines, X.-Y. Li, M. Stadler, H. Garcia, M. B. Eisen, X. Darzacq, Dense Bicoid hubs accentuate binding along the morphogen gradient. *Genes Dev.* **31**, 1784–1794 (2017).
10. A. Wodarz, Extraction and immunoblotting of proteins from embryos. *Methods Mol. Biol.* **420**, 335–345 (2008).
11. B.-C. Chen, W. R. Legant, K. Wang, L. Shao, D. E. Milkie, M. W. Davidson, C. Janetopoulos, X. S. Wu, J. A. Hammer 3rd, Z. Liu, B. P. English, Y. Mimori-Kiyosue, D. P. Romero, A. T. Ritter, J. Lippincott-Schwartz, L. Fritz-Laylin, R. D. Mullins, D. M. Mitchell, J. N. Bembenek, A.-C. Reymann, R. Böhme, S. W. Grill, J. T. Wang, G. Seydoux, U. S. Tulu, D. P. Kiehart, E. Betzig, Lattice light-sheet microscopy: imaging molecules to embryos at high spatiotemporal resolution. *Science* **346**, 1257998 (2014).
12. T.-L. Liu, S. Upadhyayula, D. E. Milkie, V. Singh, K. Wang, I. A. Swinburne, K. R. Mosaliganti, Z. M. Collins, T. W. Hiscock, J. Shea, A. Q. Kohrman, T. N. Medwig, D. Dambournet, R. Forster, B. Cunniff, Y. Ruan, H. Yashiro, S. Scholpp, E. M. Meyerowitz, D. Hockemeyer, D. G. Drubin, B. L. Martin, D. Q. Matus, M. Koyama, S. G. Megason, T. Kirchhausen, E. Betzig, Observing the cell in its native state: Imaging subcellular dynamics in multicellular organisms. *Science* **360** (2018).
13. Website. https://github.com/alecheckert/quot.
14. Website. https://github.com/scopetools/cudadecon or 10.5281/zenodo.7659013.
15. X. Ruan, M. Mueller, G. Liu, F. Görlitz, T.-M. Fu, D. E. Milkie, J. Lillvis, A. Killilea, E. Betzig, S. Upadhyayula, Image processing tools for petabyte-scale light sheet microscopy data, *bioRxiv* (2024)p. 2023.12.31.573734.
16. M. Pachitariu, C. Stringer, Cellpose 2.0: how to train your own model. *Nat. Methods* **19**, 1634–1641 (2022).
17. A. Archit, S. Nair, N. Khalid, P. Hilt, V. Rajashekar, M. Freitag, S. Gupta, A. Dengel, S. Ahmed, C. Pape, Segment Anything for Microscopy, *bioRxiv* (2023)p. 2023.08.21.554208.
18. A. Kirillov, E. Mintun, N. Ravi, H. Mao, C. Rolland, L. Gustafson, T. Xiao, S. Whitehead, A. C. Berg, W.-Y. Lo, P. Dollár, R. Girshick, Segment Anything (2023). http://arxiv.org/abs/2304.02643.
19. A. S. Hansen, I. Pustova, C. Cattoglio, R. Tjian, X. Darzacq, CTCF and cohesin regulate chromatin loop stability with distinct dynamics. *Elife* **6** (2017).
20. D. Mazza, A. Abernathy, N. Golob, T. Morisaki, J. G. McNally, A benchmark for chromatin binding measurements in live cells. *Nucleic Acids Res.* **40**, e119 (2012).
21. A. Panday, R. Elango, N. A. Willis, R. Scully, A modified CUT&RUN-seq technique for qPCR analysis of chromatin-protein interactions. *STAR Protoc* **3**, 101529 (2022).
22. P. J. Skene, J. G. Henikoff, S. Henikoff, Targeted in situ genome-wide profiling with high efficiency for low cell numbers. *Nat. Protoc.* **13**, 1006–1019 (2018).
23. S. A. Blythe, E. F. Wieschaus, Establishment and maintenance of heritable chromatin structure during early embryogenesis. *Elife* **5** (2016).
24. J. D. Buenrostro, B. Wu, H. Y. Chang, W. J. Greenleaf, ATAC-seq: A Method for Assaying Chromatin Accessibility Genome-Wide. *Curr. Protoc. Mol. Biol.* **109**, 21.29.1–21.29.9 (2015).
25. F. Ramírez, D. P. Ryan, B. Grüning, V. Bhardwaj, F. Kilpert, A. S. Richter, S. Heyne, F. Dündar, T. Manke, deepTools2: a next generation web server for deep-sequencing data analysis. *Nucleic Acids Res.* **44**, W160–5 (2016).
26. K. Bogart, J. Andrews, Extraction of total RNA from Drosophila. *CGB Tech. Rep.* **2006** (2006).
